# Mechanisms of Autoantibody-Induced Pathology

**DOI:** 10.3389/fimmu.2017.00603

**Published:** 2017-05-31

**Authors:** Ralf J. Ludwig, Karen Vanhoorelbeke, Frank Leypoldt, Ziya Kaya, Katja Bieber, Sandra M. McLachlan, Lars Komorowski, Jie Luo, Otavio Cabral-Marques, Christoph M. Hammers, Jon M. Lindstrom, Peter Lamprecht, Andrea Fischer, Gabriela Riemekasten, Claudia Tersteeg, Peter Sondermann, Basil Rapoport, Klaus-Peter Wandinger, Christian Probst, Asmaa El Beidaq, Enno Schmidt, Alan Verkman, Rudolf A. Manz, Falk Nimmerjahn

**Affiliations:** ^1^Lübeck Institute of Experimental Dermatology, University of Lübeck, Lübeck, Germany; ^2^Laboratory for Thrombosis Research, IRF Life Sciences, KU Leuven Campus Kulak Kortrijk, Kortrijk, Belgium; ^3^Neuroimmunology, Institute of Clinical Chemistry, University Hospital Schleswig-Holstein, Kiel, Germany; ^4^Neuroimmunology, Institute of Clinical Chemistry, University Hospital Schleswig-Holstein, Lübeck, Germany; ^5^Department of Neurology, University of Kiel, Kiel, Germany; ^6^Department of Internal Medicine III, University of Heidelberg, Heidelberg, Germany; ^7^Thyroid Autoimmune Disease Unit, Cedars-Sinai Medical Center, UCLA School of Medicine, Los Angeles, CA, United States; ^8^Institute for Experimental Immunology, Affiliated to Euroimmun AG, Lübeck, Germany; ^9^Department of Neuroscience, University of Pennsylvania Medical School, Philadelphia, PA, United States; ^10^Department of Rheumatology, University of Lübeck, Lübeck, Germany; ^11^Department of Dermatology, University of Lübeck, Lübeck, Germany; ^12^SuppreMol GmbH, Martinsried, Germany; ^13^Department of Neurology, Institute of Clinical Chemistry, University Medical-Centre Schleswig-Holstein, Lübeck, Germany; ^14^Institute for Systemic Inflammation Research, University of Lübeck, Lübeck, Germany; ^15^Department of Medicine, University of California, San Francisco, CA, United States; ^16^Department of Physiology, University of California, San Francisco, CA, United States; ^17^Department of Biology, Institute of Genetics, University of Erlangen-Nuremberg, Erlangen, Germany

**Keywords:** autoimmunity, autoantibodies, treatment, pathogenesis, mouse models, B cells, diagnosis

## Abstract

Autoantibodies are frequently observed in healthy individuals. In a minority of these individuals, they lead to manifestation of autoimmune diseases, such as rheumatoid arthritis or Graves’ disease. Overall, more than 2.5% of the population is affected by autoantibody-driven autoimmune disease. Pathways leading to autoantibody-induced pathology greatly differ among different diseases, and autoantibodies directed against the same antigen, depending on the targeted epitope, can have diverse effects. To foster knowledge in autoantibody-induced pathology and to encourage development of urgently needed novel therapeutic strategies, we here categorized autoantibodies according to their effects. According to our algorithm, autoantibodies can be classified into the following categories: (1) mimic receptor stimulation, (2) blocking of neural transmission, (3) induction of altered signaling, triggering uncontrolled (4) microthrombosis, (5) cell lysis, (6) neutrophil activation, and (7) induction of inflammation. These mechanisms in relation to disease, as well as principles of autoantibody generation and detection, are reviewed herein.

## Autoantibody-Mediated Diseases: One Major Medical Burden, a Congregation of Different Pathways to Disease Manifestation

Over the past decades, a sharp increase in autoimmune diseases has been noted worldwide (1, 2). The cumulative prevalence of autoimmune diseases caused by autoantibodies is well over 2.5% (3). Despite developing insights into the pathogenesis of autoantibody-mediated autoimmune diseases (reviewed herein), systemic immunosuppression, i.e., with high doses of corticosteroids, is still the backbone of the treatment. Consequently, patients suffer from a high, and partially treatment-associated, morbidity and face an increased mortality (4). Thus, there is a high, and thus far, unmet medical need for development of novel treatments for patients suffering from autoantibody-mediated autoimmune diseases.

**Figure 1:**
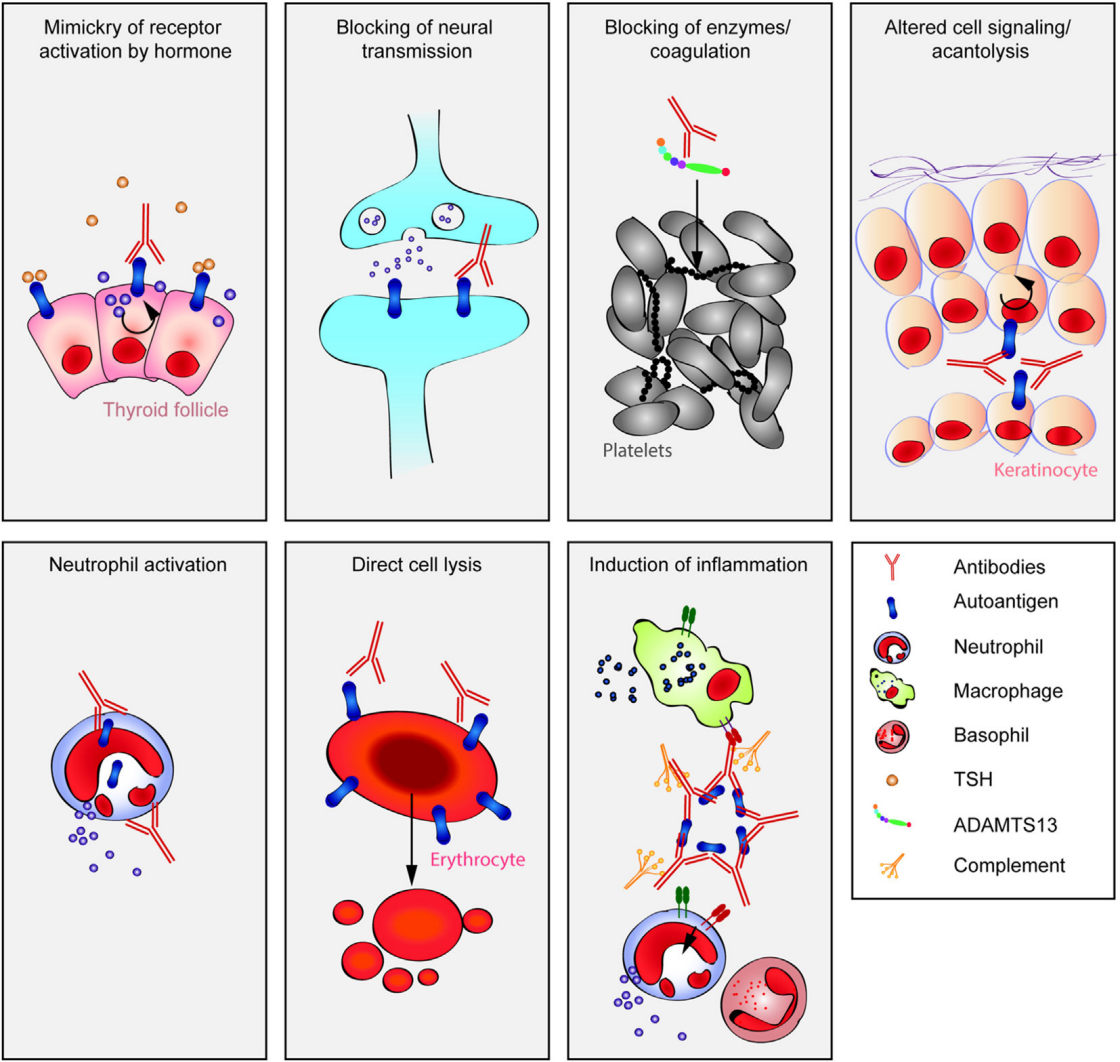
**Multiple pathways lead to autoantibody-induced pathology**. Depending on the targeted autoantigen, and sometimes even depending on the targeted epitope within a single autoantigen, autoantibodies induce pathology through specific and distinct mechanisms. Some are highlighted in this cartoon (from upper left to lower right): antibodies against the thyrotropin receptor (TSHR) mimic hormone stimulation of the TSHR receptor leading to hyperthyroidism, blockade of neural transmission by autoantibody binding to the corresponding receptors may lead to severe neurological diseases such as anti-*N*-methyl-d-aspartate encephalitis, autoantibody-mediated blockade of enzymes of the primary hemostasis may trigger uncontrolled microthrombosis, in pemphigus, autoantibodies induce an altered signaling in keratinocytes, which either reflects or leads to, a loss of cell–cell adhesion, resulting in severe skin blistering, autoantibodies to antigens expressed by neutrophils can lead to their uncontrolled activation, resulting in severe tissue injury, in autoimmune idiopathic thrombocytopenia autoantibodies trigger thrombocytopenia and severe bleeding, Fcγ-mediated functions may trigger tissue inflammation in many autoimmune diseases, e.g., rheumatoid arthritis and pemphigoid disease.

However, autoantibodies induce disease through a multitude of pathophysiological pathways. These differ among autoimmune diseases, yet within diseases multiple mechanisms may contribute to clinical manifestation. To disentangle these different autoantibody-mediated disease mechanisms, we aimed to categorize autoantibodies according to their main pathologic features. In brief, albeit surely not complete (Table 1), autoantibodies specific for a range of autoantigens induce pathology by a variety of mechanisms (Figure 1).

1. *Mimic hormone stimulation of receptor*: thyroid-stimulating autoantibodies (TSAb) in Graves’ disease.
2. *Blockade of neural transmission by receptor blockade or alteration of the synaptic structures*: antibodies against muscle nicotinic acetylcholine receptors (AChRs) in myasthenia gravis or against *N*-methyl-d-aspartate-(NMDA)-receptor in anti-NMDA encephalitis.
3. *Induction of altered signaling*: antibodies against desmoglein-3 in pemphigus.
4. *Triggering uncontrolled microthrombosis*: autoantibodies against ADAMTS13 in acquired thrombotic thrombopenic purpura.
5. *Cell lysis*: anti-platelet autoantibodies in autoimmune idiopathic thrombocytopenia.
6. *Uncontrolled neutrophil activation*: antineutrophil cytoplasmatic autoantibodies in granulomatosis with polyangitis.
7. *Induction of inflammation at the site of autoantibody binding*: autoantibodies against structural proteins of the skin in PD, or autoantibodies targeting myosin in myocarditis or autoantibodies recognizing citrullinated proteins in rheumatoid arthritis (RA) or autoantibodies targeting aquaporin-4 (AQP4) in neuromyelitis optica (NMO).

**Table 1:** **Autoantibody-mediated diseases not discussed in this review**.

Disease	Autoantibody(ies)	Pathogenic mechanism	Reference
Systemic lupus erythematosus (SLE)	Several, for example, ANA, anti-dsDNA	– Transfer of human SLE serum induces nephritis in mice – Depends on Mac-1 regulation of FcγRIIA-mediated neutrophil recruitment	(5)
Sjögren’s syndrome	Several, for example anti-Ro, anti-La, ANA	Immunization with Ro leads to a decrease in salivary flow and lymphocytic infiltration in the salivary glands	(6, 7)
Autoimmune myopathies	Several, anti-SRP, anti-HMGCR, anti-myosin	Immunization with muscle homogenisates induces autoantibodies and myositis in SJL/J mice	(8, 9)
Type I diabetes	Several, directed against insulin, glutamic acid decarboxylase and protein tyrosine phosphatase	Autoantibodies are associated with insulits and/or onset of diabetes	(10, 11)
Addison disease	Anti-steroidogenic cytochrome P450 enzyme 21-hydroxylase	– Present in patients – Induction of immune responses upon immunization in mice	(12)
Pernicious anemia	Anti-parietal cell antibodies	Reduced uptake of vitamin B12 due to lack of intrinsic factor	(13)
Autoimmune hepatitis	Several, anti-ASMA, anti-actin, ANA	– Used for diagnosis – Detection of autoantibodies in mouse models of the disease	(14)
Primary biliary cholangitis (PBC)	Antimitochondrial antibodies	– Used for diagnosis – Observed in mice with PBC	(15, 16)
Autoimmune pancreatitis	Anti-amylase α2	– Used for diagnosis – Observed in mice with the disease	(17, 18)
Goodpasture’s disease	Anti-type IV collagen antibodies (COL4)	– Immunization with COL4 induces the disease in mice – Production of autoantibodies T cell-dependent	(19)
Primary membranous nephropathy	Anti-PLA2R, anti-THSD7A	Anti-THSD7A induce proteinuria, and initiate a histopathological pattern that is typical of the disease	(19, 20)
Ovarian insufficiency	Anti-HSP90, anti-HSPA5	HSPA5 immunization induces ovarian insufficiency in mice	(21)
Autoimmune orchitis	Antisperm antibodies	Correlation of autoantibody titers with disease in mice	(22, 23)
Dry eye disease	Severla, for example, anti-kallikrein 13	– Antibody transfer induces disease – Depends on complement and Gr-1+ cells	(24)
Idiopathic interstitial pneumonias	Several	Not known so far	(25)

*In addition to the autoantibody-induced diseases covered in depth, there are several other diseases where autoantibodies can be detected and where autoantibodies are very likely to be involved in driving pathology. In most of these diseases, however, the contribution of autoantibodies is augmentation of the pathology and/or the direct contribution of the autoantibodies is less clear than in the diseases covered herein*.

With this review, we intend to foster knowledge in an increasingly important medical field. Mostly, we encourage clinicians and scientists alike to build on the current knowledge on autoantibody-mediated pathology summarized here to develop urgently needed novel treatment modalities for patients suffering from autoantibody-mediated autoimmune diseases. We begin by briefly reviewing mechanisms leading to the generation and maintenance of autoantibodies, then discuss pathophysiological pathways in relation the corresponding diseases, and close by reviewing the diagnostic tools for autoantibody detection.

## Generation and Maintenance of Autoantibodies

The presence of autoantibodies in serum reflects leakiness of central and/or peripheral tolerance mechanisms, allowing the maturation of autoantibody-producing B cells and their subsequent differentiation into antibody-secreting plasma cells. The mechanisms that normally mediate B cell tolerance include multiple selection steps that deplete or functionally silence autoreactive B cells. These well-orchestrated processes occur at the B cell immature, transitional, and mature stages (26, 27), and possibly also after activation of B cells in the periphery before they enter the plasma cell compartment in bone marrow (28, 29). The complex mechanisms contributing to B cell tolerance are multifaceted and may involve receptor editing, controlled migration, and limited availability of BAFF, CD22, Siglec-G, miRNA, and follicular regulatory T cells (30–36). These mechanisms have been extensively discussed elsewhere and are beyond the scope of this review. Nevertheless, we stress here that autoreactive B cells are selected against according to their receptor binding affinities (i.e., membrane-bound antibodies) to self-antigens. B cells producing antibodies that bind with high affinity to self-antigens are efficiently eliminated or undergo anergy, while those B cells that produce autoantibodies with medium or low binding affinity may escape, even in non-autoimmune individuals (37, 38). Many of these low-affinity autoantibodies are polyreactive and recognize self-antigens as well as pathogen-derived antigens (38, 39).

Autoantibodies are not necessarily pathogenic. Natural polyreactive/autoreactive IgM antibodies can sometimes protect from autoimmune diseases (40). There is also increasing evidence that immunoglobulin (Ig)G autoantibodies can exhibit anti-inflammatory capacities, depending on their IgG subclass (isotype) and the extent of glycosylation/sialylation of the Fc glycan linked to Asn297 (41–43). These properties modulate antibody binding to a variety of different Fc-receptors on innate effector cells (43). This receptor family includes FcγRI (CD64), FcγRIIIA (CD16a), and FcγRIIIB (CD16b) that mediate activating signals, but also includes the inhibiting receptors FcγRIIA and FcγRIIB (CD32). Antibodies exhibiting a distinct glycosylation/sialylation status and/or belonging to different IgG subclasses bind to activating and inhibiting Fc-receptors with different affinities (41, 44). Consequently, IgG subclass and glycosylation/sialylation patterns determine whether an autoantibody exhibits FcγR-mediated pro-or anti-inflammatory functions (45). Hence, differential autoantibody glycosylation might be an important regulator of autoimmune disorders (46–48).

**Figure 2:**
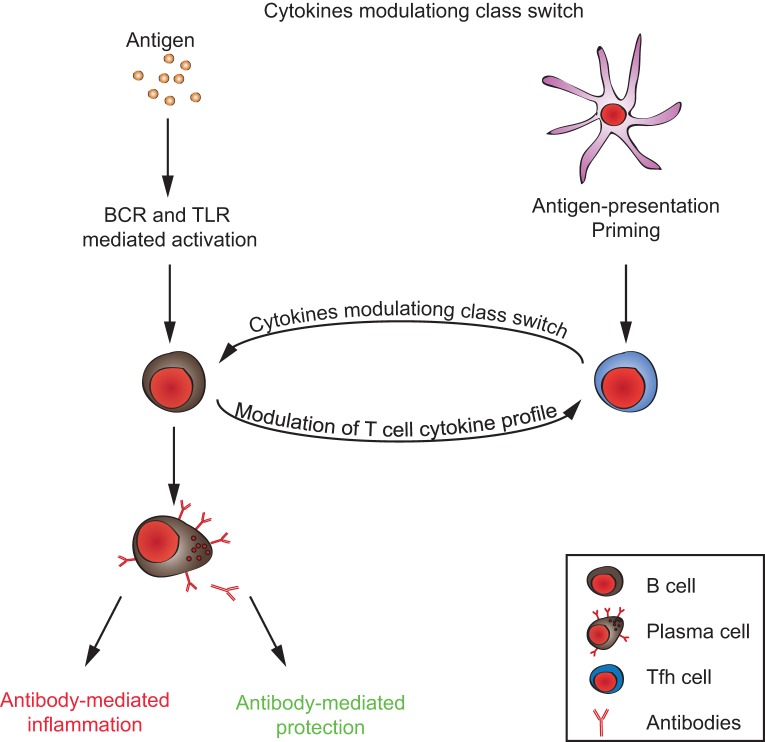
**T/B cell cross talk in generating the autoimmune response**. Autoantibodies can either promote or inhibit inflammation, depending on their immunoglobulin-isotype and glycosylation/sialylation patterns of their Fc-regions. Signals from both antigen-presenting B cells to T cells and from T cells to B cells, together, determine the inflammatory/anti-inflammatory property of the antibody response.

So far, little is known about the mechanisms that control antibody glycosylation/sialylation patterns. IgGs derived in the context of T-independent immune reactions in murine models were shown to exhibit a high degree of sialylation that mediates anti-inflammatory properties, while IgGs generated in the context of T-dependent reactions were poorly sialylated and pro-inflammatory. In mice deficient for both the IFN-γ and the IL-17 receptors, T-dependent IgGs exhibit a high degree of sialylation, suggesting that T cell-derived IFN-γ and IL-17 are involved in the regulation of antibody sialylation (49). These experiments could not formally rule out the possibility that these cytokines were derived from cells other than T cells. Nevertheless, these observations suggest that similar to what is long known for antibody class switch (50), antibody sialylation is also modulated by the interaction of activated B cells with T follicular helper cells (49). Interestingly, both T cell differentiation into follicular helper T cells and cytokine profiles are modulated by activated B cells and plasma cells themselves, *via* presentation of antigen, co-stimulatory molecules, and B cell-derived cytokines (51–56). Following TLR stimulation, B cells produce different cytokines than dendritic cells (57). Dendritic cells might be the most important antigen-presenting cells during T cell priming. However, there is evidence that later, antigen presentation by B cells is important to promote the expansion of activated T cell clones, the development of robust T effector responses, and normal T cell memory compartments (58–60). Moreover, it was shown that TLR-signals in murine B cells promote IFN-γ production from T cells and in consequence control antibody isotype switching to IgG2 *in vivo* (57). Hence, the cross talk between activated B and T lymphocytes seems to be crucial for the outcome of antibody responses and their pathogenic potential, i.e., the antibody class and glycosylation pattern (Figure 2).

Measurement of autoantibodies is a major diagnostic tool in many diseases. However, autoantibodies are often found in otherwise healthy individuals (61–64). Considering the importance of the autoantibody subclass and glycosylation pattern for the pathogenic potential of a particular antibody, it might be helpful to include these parameters into the diagnostic analysis.

Once the production of pathogenic autoantibodies has started, it could be maintained either by ongoing activation of autoreactive B cells resulting in the continuous formation of short-lived plasma cells or through the formation of long-lived plasma cells, or both (65, 66). While B cell activation and short-lived plasma cell responses are suppressed by current therapeutic treatment options, long-lived plasma cells remain a therapeutic challenge (67, 68). A novel approach to deplete long-lived plasma cells and otherwise refractory autoantibodies is treatment with the proteasome inhibitor bortezomib. This drug was shown to deplete short-lived and long-lived plasma cells in murine models of systemic lupus erythematosus (SLE) and experimental autoimmune myasthenia gravis. In these experiments, the capacity of bortezomib to suppress lupus nephritis and myasthenic symptoms has been demonstrated (69–71).

Results of the first clinical investigations using bortezomib for the treatment of refractory SLE and thrombotic thrombocytopenic purpura (TTP) are promising (72–74). However, additional controlled studies are required to elucidate the potential of bortezomib to eliminate otherwise refractory autoantibodies. Bortezomib affects neither naïve nor memory B cells. Once long-lived plasma cells are depleted, these cell types could differentiate into new plasma cells. Accordingly, bortezomib treatment is able to result in long-lasting depletion of autoantibodies only if applied in combination with B cell depletion (75, 76). The development of proteasome inhibiting drugs exhibiting fewer side effects than bortezomib might be necessary to allow application of therapeutic proteasome inhibition to a broader set of patients.

## Autoantibody-Induced Stimulation of Receptors

### Grave’s Disease

Thyroid autoimmunity involves a breakdown in self-tolerance to three thyroid proteins: thyroglobulin, thyroid peroxidase (TPO), and the thyroid-stimulating hormone receptor (TSHR) (77). Autoantibodies to TPO and/or thyroglobulin are invariably associated with Hashimoto’s thyroiditis, but with a lower prevalence in Graves’ disease. Autoantibodies that arise spontaneously to the TSHR, the direct cause of Graves’ disease, have a number of unusual properties.

**Figure 3:**
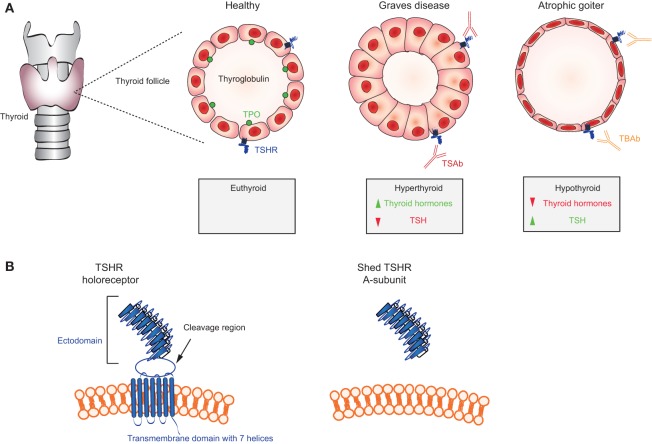
**Mechanisms in thyroid autoimmunity**. **(A)** Location of the thyroid gland in relation to the larynx and trachea and schematic representations of a thyroid follicle in a healthy individual (cuboidal cells) with the location of the thyroid-stimulating hormone receptor (TSHR), thyroglobulin, and thyroid peroxidase (TPO); a stimulated follicle in a patient with thyroid-stimulating autoantibodies (TSAb) (Graves disease; columnar epithelial cells); and a follicle in a patient with TSH blocking antibodies (TBAb) (atrophic goiter; very thin epithelial cells). Thyroid hormones (T4, T3) are elevated and TSH levels are very low in Graves’ disease; conversely, T4 and T3 are low and TSH is elevated TSH levels in atrophic goiter. **(B)** Representation of the TSH holoreceptor including its transmembrane domain (left) and the TSHR A-subunit (right) shed after cleavage.

*First*, in Graves’ disease, TSAb mimic TSH and activate the TSH receptor in an unregulated manner, thereby causing hyperthyroidism (78) (Figure 3A). Transplacental transfer of TSAb in a mother with Graves’ disease may lead to transient neonatal hyperthyroidism (79). This confirms the role of TSAb in thyroid stimulation *in vivo*. *Second*, the TSHR itself plays a role in the breakdown in self-tolerance to the receptor in genetically susceptible individuals. The highly glycosylated extracellular TSHR A-subunit, formed by intramolecular cleavage of the holoreceptor, is shed and is the autoantigen primarily responsible for TSAb induction in Graves’ disease (80–82) (Figure 3B). *Third*, in rare patients, non-activating TSHR autoantibodies can compete for TSH binding and block TSH stimulation [TSH blocking antibodies (TBAb)], thereby causing hypothyroidism with an atrophic goiter (83, 84) (Figure 3A). Hashimoto’s thyroiditis, which is a common autoimmune condition, may also lead to hypothyroidism by TPO-specific T cells and possibly TPO autoantibody-mediated damage by NK cells and activation of the complement cascade (85). *Fourth*, the assays used to measure TSHR autoantibodies for clinical purposes are unusual. Unlike most autoantibodies that can be detected by enzyme-linked immunosorbent assay (ELISA) or western blotting (see below), pathogenic TSHR antibodies are measured by the inhibition of TSH binding to its receptor (“TBI”) or in bioassays involving the generation of cAMP from monolayers of TSHR-expressing cells (“TSAb” activity) (86). *Fifth*, in experimental models, TSHR autoantibodies induced by conventional immunization using TSHR protein and a variety of adjuvants do not stimulate the thyroid gland and do not induce experimental Graves’ disease (87). Instead, TSAb and Graves’-like hyperthyroidism can be induced in mice or hamsters by injecting intact eukaryotic cells expressing the TSHR or by injecting plasmid/adenoviral vectors encoding the TSHR or, more efficiently, its A-subunit (77, 87). Valuable monoclonal antibodies (MAbs) have been isolated from mice immunized using these novel approaches (88, 89).

It should be noted that the *human* TSHR or its A-subunit are used for immunization in almost all studies. Mice immunized with adenovirus encoding *mouse* TSHR A-subunit fail to develop TSHR antibodies because of central tolerance to the endogenous TSHR. TSHR-deficient mice injected with adenovirus encoding *mouse* TSHR A-subunit develop TSHR antibodies (90). Adoptive transfer of splenic T cells from immunized TSHR-deficient donors to wild-type recipients on the same genetic background reveals the stimulatory ability of these induced TSHR autoantibodies (91). Finally, although TSHR autoantibodies are unusual, Graves’ hyperthyroidism is a common condition, with a prevalence of ~1% (92). Moreover, TSHR autoantibodies play a role in Graves’ exophthalmos and Graves’ dermopathy by interacting with the TSHR expressed on orbital fibroblasts (93) and dermal fibroblasts (94). It is notable that patients with Graves’ exophthalmos and dermopathy usually have very high circulating levels of TSHR autoantibodies (94).

#### Properties of TSHR Autoantibodies

The immunological properties of TSAb and TBAb, based on patients’ sera and human MAbs derived from Graves’ patients, are described in detail elsewhere (95, 96). In brief, TSAb are present at very low (ng/ml) concentrations in serum (97–99). In contrast, TBAb are present at much higher concentrations, μg/ml (80, 99). TSHR autoantibodies are predominantly IgG although IgA- and IgE-class TSHR autoantibodies have been observed by flow cytometry (100). Both serum TSHR autoantibodies and human mAb have extremely high affinities, as would be expected for antibodies that compete with TSH for binding to its receptor. “Switching” from TBAb to TSAb (or *vice versa*) is a rare phenomenon observed in some patients. These changes involve differences in the concentrations of TSAb versus TBAb, as well as their affinities and/or potencies in individual patients (86). Anti-thyroid drugs or immunosuppression/hemodilution in pregnancy reduce initially low TSAb levels even further while higher concentration TBAb may persist. In contrast, if TSAb emerge during levothyroxine administration for TBAb-induced hypothyroid, these stimulating antibodies may be sufficient to swing the pendulum to hyperthyroidism.

Thyroid-stimulating autoantibodies and TBAb interact with conformational epitopes and do not bind to synthetic TSHR peptides. Crystallization of both types of mAb with recombinant TSHR-A-subunit protein reveal closely overlapping epitopes with subtle differences expected for a TSAb (M22) and a TBAb (K1-70) (101, 102). Unlike spontaneously arising TSHR autoantibodies, some TSHR mAb derived from immunized hamsters bind to a linear peptide in the region deleted from the single chain TSH holoreceptor after intramolecular cleavage into A- and B-subunits. These “neutral” or “cleavage region” TSHR antibodies neither stimulate the thyroid nor block TSH stimulation but may play a role in signaling cascades leading to apoptosis (103). Because the epitope of these neutral antibodies (unlike TSAb and TBAb) is lost after TSHR cleavage into subunits, they can only interact with the single chain, uncleaved TSHR. Whether the TSHR *in vivo* exists in the uncleaved form has been the subject of debate (82). In human disease, confirmation of the existence of neutral TSHR autoantibodies will require information on their concentrations or affinities. To date, no human “neutral/cleavage” TSHR autoantibodies have been cloned.

#### Current and Future Approaches to Treat Graves’ Disease

Several approaches are available to treat the hyperthyroidism in Graves disease including thionamide drugs, thyroid ablation by Radioiodine (131-I), or subtotal thyroidectomy as well as β-blockers to inhibit the actions of excess thyroid hormone (104). In general, these therapies are effective, thionamide drugs are inexpensive, and all approaches have been used for many years. Radioiodine therapy frequently leads to permanent hypothyroidism, subsequently leading to life-long thyroid hormone replacement. Rarely, development of serious side effects with thionamide drugs requires one of the alternative forms of treatment. Atrophic goiter is treated by replacement of thyroid hormone (levothyroxine, LT4), which is safe and inexpensive.

Three novel forms of therapy for Graves’ hyperthyroidism are currently under investigation: (i) small molecule inhibitors of TSHR function (105–107), (ii) monoclonal TBAb, such as K1-70 (96) that block the binding and function of TSAb, and (iii) inhibitors of components of the adaptive immune system, such as rituximab, which targets B-lymphocytes prior to their maturation into plasma cells (Table 2).

**Table 2:** **Selected drugs targeting autoantibody-induced pathology**.

Drug/intervention	Phase	Target/mode of action	Indications(s)	Reference
Anti-CD20 (Rituximab)	Clinical use	CD20 (B cells)	– Grave’s disease – SSc – TTP – Vasculitis – Pemphigus	(108) (109)
Immunadsorption/plasmapheresis	Clinical use	Temporary removal of all antibodies	– MG – Pemphigus – Pemphigoid – TTP – Autoantibody-induced carditis	(110)
IVIG	Clinical use	Several, inhibition of the FcRn reduced circulating autoantibodies	– MG – Autoantibody-induced carditis – Pemphigus – NMOSD	(111)
Caplacizumab	Clinical use	Anti-von Willebrand nanobody	– TTP	(112)
Eculizumab	Off label use	C5	– Licensed for paroxysmal nocturnal hemoglobinuria – Proof-of-concept study in NMOSD	(113)
SHP652 (SM101)	Phase II	Fc/FcγR interactions	– Systemic lupus erythematosus – Idiopathic thrombocytopenic purpura	(114)
R935788	Phase II	SYK	– Rheumatoid arthritis – Pemphigoid (preclinical)	NCT00665626 (115)
K1-70	Phase I	Thyroid-stimulating hormone receptor antagonist	Grave’s disease	(116)
BAX930	Phase I	BAX is recombinant ADAMTS13, given to reconstitute ADAMTS13	– TTP	NCT02216084
CAAR T cells	Preclinical	Autoantigen-specific B/plasma cells	– Pemphigus	(117)
EGFR inhibition	Preclinical	EGFR	– Pemphigus	(118)
Tandem peptide	Preclinical	Tandem peptide consisting of 2 connected peptide sequences targeting the desmoglein-3	– Pemphigus	(119)
Apremilast	Preclinical	PDE4 Inhibition	– Licensed for psoriasis – Preclinical evidence for pemphigoid	(120, 121)

*SSc, systemic sclerosis; MG, myasthenia gravis; IVIG, intravenous immunoglobulin; PDE4, phosphodiesterase 4; FcRn, neonatal Fc-receptors; TTP, thrombotic thrombocytopenic purpura; NMOSD, neuromyelitis optica spectrum disorder; SYK, spleen tyrosine kinase; CAAR, chimeric autoantigen receptor T cells; EGFR, epidermal growth factor receptor*.

*References to websites were all accessed on April 28, 2017*.

Small molecule inhibitors and monoclonal TBAb target the proximate cause of hyperthyroidism, the TSHR. Both of these new forms of therapy, if successful, are likely to be more expensive than the presently available and effective therapies. In addition, because small molecule inhibitors interact with the heptahelical transmembrane domain of the TSHR, this treatment option carries the risk of side effects consequent to potential cross-reactivity with others in the very large G-protein-coupled receptor family. Graves’ ophthalmopathy occurs with varying degrees of severity and can be very distressing symptomatically and cosmetically, as well as (fortunately rarely) leading to loss of vision. Options for the treatment of Graves’ ophthalmopathy include, in addition to the restoration of the euthyroid state, corticosteroids, orbital irradiation, and orbital surgical decompression. Because none of these therapeutic modalities are optimal, there is presently much interest in the use of antibodies to CD20 (rituximab) that cause B cell depletion. Two recent double-blind clinical trials with rituximab have been reported: in patients with long-standing Graves’ eye disease (10 months), rituximab was no more effective than control saline injections (122). In contrast, in patients in whom eye disease duration was short (4.5 months), rituximab was more effective at reducing clinical symptoms than methylprednisone (123). The different outcomes of these two studies emphasize the importance of early intervention, which, in turn, requires early disease diagnosis.

The challenge in Graves’ disease is to specifically inhibit the development, or ongoing production, of TSHR autoantibodies. Such an approach has been attempted in mouse models of induced Graves’ disease. First, injecting neonatal BALB/c mice with TSHR A-subunit protein induced tolerance that could not be broken by subsequent immunization with adenovirus encoding A-subunit (124). Second, hyperthyroidism was attenuated in adult mice by injecting TSHR A-subunit protein before A-subunit adenovirus immunization. The latter involved deviating pathogenic antibodies to non-functional antibodies and was only effective if applied before immunization, not after hyperthyroidism had been established (125). However, in a mouse model that spontaneously develops pathogenic TSHR antibodies (126), injecting TSHR A-subunit protein failed to divert the autoantibody response to a non-pathogenic form, highlighting critical differences between induced and spontaneous Graves’ disease models, with implications for potential immunotherapy in humans (127).

### Systemic Sclerosis (SSc)

Systemic sclerosis or scleroderma is one of the most lethal rheumatic diseases characterized by microvascular dysfunction, dysregulation of innate and adaptive immunity, and interstitial and perivascular fibrosis in the skin and internal organs (128). Mechanistically, a hallmark SSc feature is the presence of high serum concentrations of multiple autoantibodies (129). Among these autoantibodies, increased titers of stimulating autoantibodies targeting both angiotensin II type 1 receptor (AT1R) and endothelin-1 type A receptor (ETAR) have been reported to contribute to SSc pathogenesis and suggested as biomarkers for risk assessment of disease progression (130). These antibodies can be detected by ELISA (see below). Anti-AT1R and anti-ETAR are strong predictors of digital ulcers development (131), they can be considered as predictive and prognostic biomarkers of (SSc)-associated pulmonary arterial hypertension (132) and are linked to the development of lung fibrosis and vasculopathies in patients with SSc (133). Data from mice models show that anti-AT1R and anti-ETAR antibody-positive SSc-IgG cause structural alterations of the lungs including induction of interstitial lung disease and obliterative vasculopathy, increased cellular density, and enhanced interstitial cellular infiltrations following passive transfer of IgG from SSc patients to wild-type mice (134, 135).

**Figure 4:**
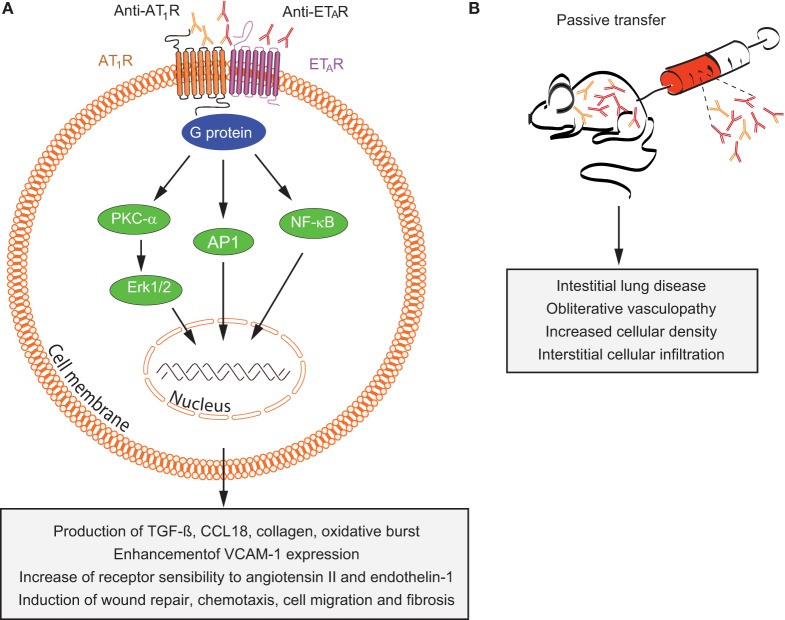
**Overview of mechanisms by which anti-AT1R and anti-ETAR induce systemic sclerosis (SSc) pathogenesis**. **(A)** *In vitro* and **(B)** *in vivo* effects of autoantibodies targeting ETAR and AT1R in SSc are shown. Abbreviations: AT1R, angiotensin II type 1 receptor; ETAR, endothelin-1 type A receptor; PKC-α, protein kinase C-α; ERK1/2, extracellular signal-regulated kinase 1/2; NF-κB, nuclear factor-κB; AP-1, activator protein 1; TGF-β, transforming growth factor β; CCL18, chemokine (C-C motif) ligand 18; VCAM-1, vascular cell adhesion molecule-1.

Autoantibodies targeting ETAR and AT1R increase the sensitivity of both AT1R and ETAR to their natural ligands (angiotensin II and endothelin-1) and have synergistic effects in the presence of these ligands (132, 136). Anti-AT1R and anti-ETAR autoantibodies activate signaling molecules and transcription factors such as protein kinase C-α (PKC-α), extracellular signal-regulated kinase 1/2, nuclear factor-κB (NF-κB), and activator protein 1, which are involved in several pathophysiologic processes (130, 136–138). Increased number of reports have recently linked a variety of anti-AT1R and anti-ETAR cellular and systemic effects to SSc pathogenesis (139). Among them, anti-AT1R and anti-ETAR from SSc patients (SSc-IgG) contribute to vasculopathies by regulating cell migration through interleukin-8 (IL-8) production and expression of vascular cell adhesion molecule-1 by human microvascular endothelial cells (HMEC-1). Consistent with these findings, IL-8 release by PBMCs, regulation of wound repair, and T-cell chemotaxis have been also observed following IgG-induced ETAR and AT1R activation (133, 134). Anti-AT1R and anti-ETAR autoantibodies directly induce collagen production by skin fibroblasts (134) and regulate the synthesis of profibrotic factors such as transforming growth factor β (TGF-β) by human dermal microvascular endothelial cells (130), which are pathological mechanisms in SSc patients who develop lung fibrosis. Another important mechanism of autoantibody-induced pathology triggered by anti-AT1R and anti-ETAR is the production of chemokine (C-C motif) ligand 18 (CCL18) (133). CCL18 levels in bronchoalveolar fluid and sera directly reflect pulmonary fibrotic activity and are predictive for lung disease progression and mortality in patients with SSc (140–142). Additional pathological mechanisms triggered by anti-AT1R and anti-ETAR have been reported such as the increase of neutrophil migration and reactive oxygen species (ROS) production by neutrophils (134). An overview of the pathological mechanisms triggered by anti-AT1R and anti-ETAR autoantibodies is shown in Figure 4.

In addition to the levels of anti-AT1R and anti-ETAR autoantibodies, AT1R and ETAR expression needs to be considered in SSc pathogenesis. The cellular effects triggered by anti-AT1R and anti-ETAR autoantibodies depend on the AT1R and ETAR expression levels. For instance, imbalanced AT1R/AT2R and ETAR/ETBR expression ratios in SSc influence autoantibody-mediated effects such as secretion of profibrotic CCL18 (143). So far, a therapy that specifically inhibits anti-AT1R and anti-ETAR autoantibodies or/and regulates AT1R/AT2R and ETAR/ETBR expression is not available. Expansion of the currently state of knowledge about the cell signaling pathways and mechanism of autoantibody-induced pathology triggered by anti-AT1R and anti-ETAR will be essential to develop new therapies specifically targeting autoantibody effects simultaneously maintaining the renin–angiotensin and endothelin systems physiological response. Achieving this goal will be essential for the management of SSc patients in order to improve their quality of life and reduce SSc mortality rates. This achievement will also provide new therapeutic options for other diseases in which high circulating levels of anti-AT1R and anti-ETAR autoantibodies have pathological effects (138, 144–148).

## Disruption of Neural Transmission by Autoantibodies

### Myasthenia Gravis (MG)

Myasthenia gravis is an uncommon autoimmune disorder characterized by muscle weakness and abnormal fatigability that worsens with use of affected muscles and improves with rest. It is caused by the presence of autoantibodies to components of the postsynaptic muscle endplate localized at the neuromuscular junction (149, 150). The annual incidence of MG is approximately 1.7–34 per million, and the prevalence ranges from 15 to 320 per million population (151–153). However, MG remains underdiagnosed and the prevalence shows a steady increase over time, which is likely reflective of better recognition of the condition, aging of the population, and the longer life span of patients, possibly due to improved disease treatment.

Myasthenia gravis is a chronic affliction that affects people of all ages and both sexes (154). Studies of large groups of patients show that the age of onset is characterized by a bimodal distribution with female predominance in the second to third decade of life and slight male predominance in the sixth to eighth decades. MG, especially early-onset MG, is often associated with other autoimmune diseases, most commonly autoimmune thyroid disease, SLE, RA, Addison’s disease, Guillain–Barré syndrome, type 1 diabetes mellitus, and NMO (155).

Several subgroups of MG have been identified based on clinical presentation, age of onset, autoantibody profile, and thymic pathology. Over two-thirds of all patients with MG begin with symptoms relating to their eye muscles (156). The symptoms usually progress to other muscles during the first 2 years, resulting in generalized MG. In about 15% of MG patients, symptoms are restricted to the eye muscles, and this condition is termed ocular MG. In most cases, a specific cause of MG cannot be identified (157). There is strong evidence that the pathogenesis of MG involves a combination of multiple genotypes of low penetrance and largely unidentified environmental factors (158). Patients with early-onset MG are usually associated with HLA-B8DR3 and thymic hyperplasia (155, 159). Approximately 15% of patients with MG have a thymoma, and 50% of thymoma patients develop MG (160). Improving the clinical symptoms by thymectomy in some MG patients, although questioned by many, also suggests a specific role of the thymus in MG (161, 162).

About 90% of generalized MG is caused by pathogenic autoantibodies to muscle nicotinic AChRs. MG patients who do not have detectable autoantibodies to AChRs are referred to as seronegative. For many years, the cause of MG remained a mystery. In 1960, Simpson suggested that MG was caused by autoantibodies to AChRs acting as competitive antagonists (163). In 1973, Patrick and Lindstrom demonstrated that MG is an autoimmune disease by showing that rabbits immunized with muscle-like AChR purified from fish electric organ developed MG-like symptoms (164). Experimental autoimmune MG (EAMG) in rabbits, was reproduced in other species, especially inbred Lewis rats where the most detailed studies have been completed (165). EAMG shares many clinical and immunopathological features with those of MG. Clinical, electrophysiological, histological, pharmacological, and immunological analysis of EAMG led to extensive understanding of the pathological mechanisms in MG (150, 166). These findings promoted the use of immunosuppressive treatments in MG.

The pathogenic role of autoantibodies to AChRs is clearly established. Most patients with MG have circulating antibodies to AChR (167). Injection of patients’ IgG or isolated autoantibodies to AChR from patients into laboratory animals passively transfers several features of MG from human to the recipients (168, 169). Immune complexes (IgG and complement) co-localize with AChRs on the postsynaptic membrane (170, 171). Plasmapheresis that removes circulating antibodies leads to a substantial, but temporary, improvement in muscle function (172). EAMG can be induced by immunization with purified AChR (164) or by injection of mAbs to AChR (173). However, among MG patients, there is not a close correlation between the absolute concentration of autoantibodies to AChR and severity (167, 174). Variability in specificities, affinity, and isotypes of the autoantibodies to AChR may contribute to this lack of correlation (175).

**Figure 5:**
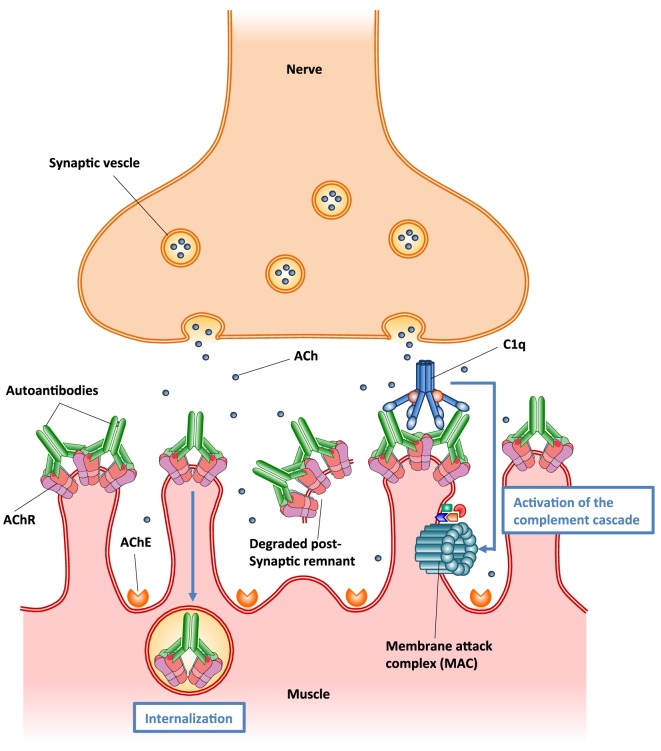
**Schematic illustration of the two major pathogenic mechanisms of autoantibodies to acetylcholine receptor (AChR)**. (1) Autoantibody binding to the AChR on the surface of postsynaptic muscle membrane activates the complement cascade, resulting in the formation of membrane attack complex (MAC) and localized destruction of the postsynaptic membrane. The immune assault releases shedding of membrane fragments containing AChRs into the synaptic cleft, and leads to a simplified, altered morphology of the postsynaptic membrane. (2) Autoantibodies cross-link AChR molecules on the postsynaptic muscle membrane, causing endocytosis of the cross-linked AChR molecules and their degradation (antigenic modulation). This leads to a reduced number of AChR molecules on the postsynaptic membrane.

Autoantibodies to AChR can differ in their isotype, affinity, and specificity to various AChR epitopes. Pathological autoantibodies are directed at conformation-dependent extracellular epitopes of AChRs, especially the main immunogenic region (MIR) on AChR α1 subunits (176). Half or more of the autoantibodies to AChR in MG target the MIR. These autoantibodies impair neuromuscular transmission primarily by two mechanisms: (1) focal complement-mediated lysis of the postsynaptic membrane that destroys AChRs and disrupts synaptic morphology (166, 171), (2) cross-linking AChRs by the autoantibodies on the surface of postsynaptic membrane accelerating endocytosis and lysosomal destruction of AChRs, termed antigenic modulation (177) (Figure 5). Some autoantibodies inhibit AChR function by direct blockage of acetylcholine (ACh) binding sites, but these appear to play a minor role (178). No cellular infiltration at the target muscle tissue of MG patients indicates that tissue damage is not caused by direct harmful effects of cytotoxic cells (170). However, autoreactive T cells provide help to B cells that produce autoantibodies to AChR (179). Autoantibodies directed at cytoplasmic epitopes of human AChR are also present in MG sera (180). However, these autoantibodies are not pathogenic because the cytoplasmic domain of AChR is not accessible to these autoantibodies in intact muscle. Additionally, rats repeatedly immunized with the cytoplasmic domains of AChR in adjuvant do not develop EAMG and serum antibodies from these rats do not bind the MIR, passively transfer EAMG, or cause antigenic modulation of AChR, although these antibodies bind to solubilized native AChRs (175, 181).

In MG patients, the predominant isotypes of autoantibodies to AChR are IgG1 and IgG3, which fix complement (182). A series of studies have suggested that accumulation of autoantibodies to AChR and subsequent activation of the complement cascade on postsynaptic membrane result in the assembly of membrane attack complex (MAC), thereby destroying AChRs and disrupting synaptic morphology (166, 171, 183) (Figure 5). It seems likely that complement-mediated destruction of the postsynaptic muscle membrane is more pathologically significant than antigenic modulation in MG pathogenesis (175, 184). These findings have promoted the development of novel therapies based on inhibition of complement pathways in MG treatment (185).

Half of AChR seronegative patients have antibodies to muscle-specific kinase (MuSK), which is a component of the agrin receptor complex that mediates clustering and stabilization of AChR in developing muscle (186). Autoantibodies to MuSK and those to AChR rarely coexist in the same patient. MuSK-related MG differs from AChR-related MG primarily by demonstrating more focal involvement and wasting of the involved muscles (187). This type of MG is predominantly in females and usually reported in adults. There is a HLA association with HLA-DQ5 (188). No thymus pathological changes have been reported and patients usually have no response to thymectomy (189). Autoantibodies to MuSK are mainly of the IgG4 isotype, which does not activate complement, and the IgG4 fraction alone is sufficient for passive transfer of the disease (188). Autoantibodies to MuSK directly suppress the postsynaptic tyrosine kinase pathway thus indirectly reduce the metabolic stability of endplate AChRs (190). Complement is not necessary for pathogenesis in mice (191). Patients with MuSK-related MG respond better to plasmapheresis than to intravenous immunoglobulin (IVIG) (192). Rituximab appears to be more effective in patients with MuSK-related MG than in patients with AChR-related MG (193).

Low-density lipoprotein receptor-related protein 4 (LRP4), which belongs to a family of the low-density lipoprotein receptor family, is a receptor for nerve-derived agrin and an activator of MUSK, and is necessary to maintain AChR function (194). Autoantibodies to LRP4 have been detected in some of double-seronegative MG patients who do not have antibodies to AChR or MuSK (195, 196). LRP4-related MG has a female preponderance. Most of these patients present with ocular or generalized mild MG. This type of MG appears to be clinically similar to AChR-related MG, but there is no clear role for thymectomy. Autoantibodies to LRP4 are predominantly of the IgG1 isotype, which fixes complement, and thus may damage the postsynaptic muscle membrane by a similar mechanism as those to the AChR. Inhibition of argin-induced AChR clustering may also be a potential pathophysiological mechanism (197).

Some seronegative patients have low-affinity antibodies to AChR that are not detectable by the classical immunoprecipitation assay. These autoantibodies can be identified by cell-based assay (CBA) only (198). These patients are similar to patients with AChR-related MG with respect to their clinical presentation, their response to treatment, and their thymic abnormalities. Some seronegative patients probably have pathogenic antibodies to as-yet-unidentified antigens in the postsynaptic membrane. Antibodies to agrin and cortactin are often identified in combination with other autoantibodies (199). Their contribution to MG pathogenesis is still unclear. Some patients with AChR-related MG have antibodies to titin and ryanodine receptor (200). These antibodies cannot enter the muscle cell *in vivo* and thus might not cause any muscle weakness, but rather could be markers of the autoimmune disease directed at muscle surface resulting in exposure of cytoplasmic proteins.

Treatment options available today rely primarily on a combination of symptomatic therapies and general, non-specific immunosuppression. Many MG patients respond favorably to treatment (149). Symptomatic treatment of MG with acetylcholinesterase inhibitor temporarily enhances neuromuscular transmission by increasing the availability of ACh to compensate for loss of AChRs, but does not induce complete or sustained relief of MG symptoms in most patients and does not alter disease progression (149, 154). Patients with MuSK-related MG may not respond to cholinesterase inhibitor. At high doses, cholinesterase inhibitor may actually cause more muscle weakness through desensitization of AChRs (201). Most patients require additional immunosuppressive treatment. High doses of corticosteroids and chronic treatment with non-specific immunosuppressive drugs are usually required to maintain disease control. Non-specific immunosuppressive drugs primarily suppress lymphocyte activation and proliferation and have little effect on long-lived plasma cells that are terminally differentiated cells and continue producing pathogenic antibodies (202). Therefore, the use of these drugs is often hampered by delayed clinical response. Plasmapheresis and IVIG are used for acute severe exacerbations in generalized MG. For a chronic disease like MG, the current treatment has a high cost (203), as most patients during the long-term treatment suffer several undesirable side effects (204). A distinct subset of patients often referred to as having refractory MG, do not respond well to current treatments (205).

Advances in understanding of the pathogenesis of the various forms of MG and breakthrough use of mAbs specific for different aspects of the immune system involved in antibody production and antibody-mediated tissue damage provide an opportunity to develop more specific treatment with long-lasting benefits and fewer adverse effects. Rituximab, a chimeric murine-human mAb that depletes B cells by binding to their CD20 surface marker, has been reported to be effective in patients with severe or refractory MG, especially in those with MuSK-related MG (193, 205). Yale University is sponsoring a multicenter clinical trial (NCT02110706) to determine whether rituximab is a safe and beneficial therapeutic for MG. BAFF is a potent survival factor for B cells and belimumab, a human mAb targeting BAFF, reduces B cell activation and differentiation into antibody-producing plasma cells. A phase II study of belimumab in AChR- and MuSK-related MG is in progress (NCT01480596). A placebo-controlled phase II study in patients with refractory generalized MG has shown that eculizubmab, a humanized mAb against C5 complement, is effective, promoting an ongoing phase III study (NCT01997229). Additionally, a trial of hematopoietic stem cell therapy in MG patients is in progress (NCT00424489).

**Figure 6:**
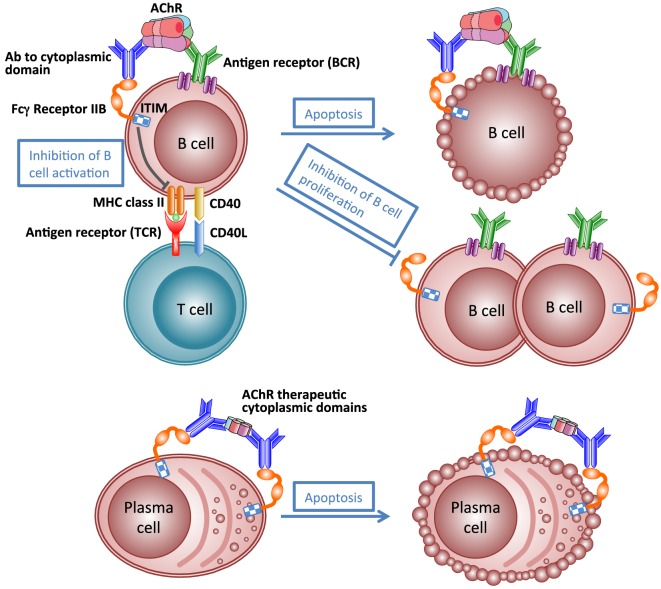
**Schematic illustration of mechanism of antibody-mediated feedback suppression**. Cross-linking of the B-cell receptor and the inhibitory IgG receptor (FcγRIIB) on the B cell surface by antigen–antibody complex may result in apoptosis of antigen-specific B cells, inhibition of B cell activation by helper T cells, and inhibition of B cell proliferation. Cross-linking FcγRIIB on the surface of plasma cells by immune complexes induces apoptosis of plasma cells.

Like many autoimmune diseases described in this review, MG is an ideal disease for antigen-specific immunotherapy because of its clearly defined autoantigen. Theoretically, an antigen-specific immunotherapy would eliminate the pathogenic autoimmune response to autoantigen specifically without affecting the other functions of the immune system, and thus avoiding severe adverse effects. Such antigen-specific immunosuppressive therapy is not yet available for MG (206). The idea of specific immunosuppression of autoimmune response to AChR by administration of AChR peptides or fragments has been investigated in EAMG for many years (181). However, translation into the clinic has been hampered by concerns about the potential for exacerbation of pathogenic autoimmune response, because the rationale behind these studies is based on a hypothesis that specific immunosuppression of autoimmune response requires the use of disease-inducing sequences (207). Recently, a vaccine using AChR cytoplasmic domains has been shown to be effective at specifically suppressing EAMG (175, 181, 208). Immunization with the vaccine in adjuvant prevents development of chronic EAMG, rapidly inhibits established EAMG, and prevents reinduction of EAMG for at least 6 months. Therapeutic effects might result from inhibition of production of pathological antibodies by a combination of antibody-mediated feedback suppression (Figure 6) and regulatory T cell-mediated active suppression. These studies indicate that not all autoantibodies are harmful. Autoantibodies to AChR cytoplasmic domain may play a therapeutic role in the antigen-specific immunotherapy. This approach may be applicable to other antibody-mediated autoimmune responses to other transmembrane proteins.

### Anti-NMDA Encephalitis

The recently discovered anti-*N*-methyl-d-aspartate-receptor (NMDAR) encephalitis—is the most common antibody-associated acute autoimmune encephalitis (209). Incidence has been estimated at 3–5/1.000.000/year (210). Although a rare disease, its description has greatly influenced neuroimmunology and neuroscience. It represents a model disease for a group of syndromes characterized by detection of autoantibodies targeting synaptic structures, hence called synaptic encephalitis (211).

Anti-NMDAR encephalitis preferentially occurs in young adults and children, predominantly women (80%). Approximately 70% of the patients develop prodromal symptoms, e.g., headache or fever, followed by rapid change of behavior including anxiety, hallucinations, and psychosis. Abnormal movements (orofacial dyskinesias, chorea, stereotyped movements) and eventually decrease of consciousness, coma, and severe global autonomic dysregulation (sometimes leading to hypoventilation and asystolia) ensue. Seizures and status epilepticus may occur at any stage of the disease. Brain MRI is normal in most but cerebrospinal fluid (CSF) shows non-specific inflammatory changes in almost all cases. Around 40% of the patients have an underlying neoplasm. The majority of tumors are ovarian teratomas. Approximately, 50% of patients respond well to IVIGs, steroids, or plasma exchange and the other 50% require rituximab alone or combined with cyclophosphamide. During recovery and after the acute symptoms have resolved, patients continue with deficits of memory and attention, impulsivity, behavioral disinhibition, and executive dysfunction that usually improve over many months. However, in some patients, recovery is incomplete, may take years, and mortality due to intensive care complications can be as high as 7% (212–214).

Diagnosis of anti-NMDAR encephalitis relies on the detection of IgG autoantibodies targeting a highly conserved and very restricted epitope on the aminoterminal-domain of the GluN1 subunit of the heterotetrameric ionotropic glutamate receptor NMDAR in patients’ CSF and serum by indirect fluorescence or ELISA (212, 215). A strong line of experimental and clinical evidence has been gathered, showing that NMDAR autoantibodies directly—without any other humoral or cellular components of the immune system involved—and reversibly interfere with NMDAR mediated synaptic function and disrupt central nervous system network function.

**Figure 7:**
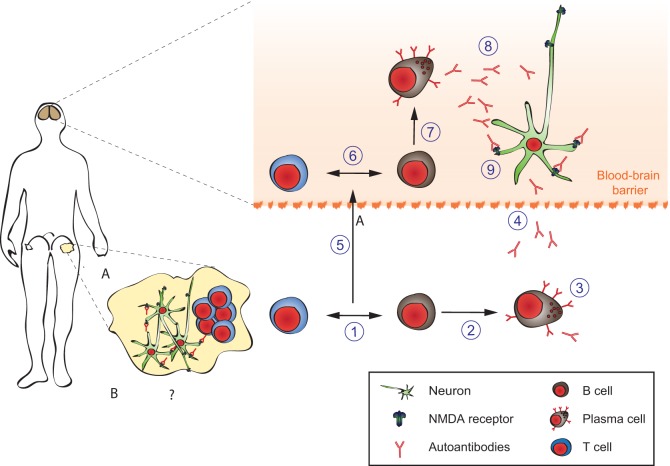
**Pathophysiology of anti-*N*-methyl-d-aspartate-receptor (NMDAR) encephalitis**. Details are explained in the text, where the numbering corresponds to the labeling used here. In brief, ectopic expression of NMDA receptors in ovarian teratomas together with co-stimulatory signals **(A)** or unknown triggers **(B)** lead to systemic immune response and formation of antigen-specific circulating B-, T-, and plasma cells producing NMDAR antibodies (1–4). The latter do not reach neurons in sufficient concentratiosn to exert effects due to the blood–brain barrier (dashed line). An unknown secondary trigger (e.g., systemic infections, 5) eventually mediates transition of B-, T-, and plasma cells into the brain. The resulting antibody production effectively increases intrathecal NMDAR antibody concentration above a threshold overwhelming neuronal compensation mechanisms and leading to net loss of surface NMDAR resulting in neurological symptoms. Adapted from Moscato et al. (216).

The pathophysiology of anti-NMDAR encephalitis can be divided into (1) initiation of systemic immune response, (2) propagation of antibody-producing cells into the central nervous system, and (3) effects of the antibodies on synaptic function (Figure 7).

(1) In paraneoplastic anti-NMDAR encephalitis, ovarian teratomas show ectopic expression of NMDAR and a dense infiltrate of T-, B-lymphocytes, and macrophages together with complement deposition in the tumors (212). The tumor-induced inflammation likely provides co-stimulation to autoreactive NMDAR specific T- and B-lymphocytes causing a breach of endogenous tolerance, systemic immune response, generation of plasma cells, and autoantibodies (Figure 7, steps 1–3). However, not all ovarian teratomas induce NMDAR antibodies presumably due to tumor-intrinsic and host-intrinsic factors (e.g., MHC class II haplotypes) (Figure 7A) (217). The initial trigger in idiopathic cases is currently unknown (Figure 7B). Interestingly, HSV-1 encephalitis can induce secondary anti-NMDAR encephalitis (218–222), and further post-infectious encephalitis variants are emerging (e.g., Japanese B-encephalitis). Furthermore, idiopathic anti-NMDAR encephalitis appears to occur seasonally in children (223). Thus, it is intriguing to speculate that in some “idiopathic” cases, yet unidentified viral infections might prove to be a trigger of anti-NMDAR encephalitis.
(2) A systemic NMDAR-directed immune response by itself does not appear to be sufficient to cause symptoms (Figure 7, step 4). Serum NMDAR antibodies can persist long after symptoms have resolved and serum titers do not correlate well with symptoms (224). The intact blood–brain barrier (BBB) prevents systemic antibodies and complement from reaching the synapse [CNS IgG levels are 400-fold lower than in serum (225)]. However, CSF NMDAR titers are correlated with disease activity (224). They are produced by dense infiltrates of plasma cells found perivascular in the CNS (Figure 7, steps 6–8) (226). The driving force behind establishing these CNS-local plasmablasts and -cells is unknown. A “second hit” hypothesis postulates a secondary infectious or inflammatory condition attracting preexisting NMDAR-antibody-producing cells to the CNS (Figure 7, step 5). Some support to this hypothesis is provided by detection of high levels of the B-cell-attracting chemokine CXCL13 in the CSF of anti-NMDAR encephalitis patients (227). Other chemokines and cytokines are also likely to be involved. Elucidation of the responsible “second hit,” the chemokines involved and the possible contribution of T-lymphocytes and long-lived plasma cells hidden behind the BBB would provide a new angle and rationale for treatment of patients, e.g., IL6-directed treatment or proteasome inhibitor treatment. It might furthermore help to explain the beneficial effects of the CD20-antibody rituximab in this condition in spite of its inability to cross the intact BBB.
(3) For anti-NMDAR encephalitis, a direct pathogenic role of GluN1 IgG antibodies has been established in cultures of neurons and after cerebroventricular infusion of patients’ antibodies to rodents (Figure 7, step 9). The antibodies are mainly composed of complement-fixing IgG1 and IgG3 isotypes, yet complement deposition is not a pathological feature of the disease (228–230). This is likely due to the low concentration of complement components in the CSF. Using cultured neurons, patients’ antibodies cause cross-linking and selective internalization of NMDARs that correlate with the antibody titers. These effects were reversible after removing the antibodies and dependent on the cross-linking ability but not the Fc-terminus of the antibodies (230). In contrast to the intense effects on NMDAR, patients’ antibodies did not alter the localization or expression of other synaptic proteins, number of synapses, dendritic spines, dendritic complexity, or cell survival (216, 231, 232). A transfer murine model using continuous ventricular infusion (14 days *via* osmotic pumps) of CSF from patients with anti-NMDAR encephalitis showed profound effects on memory and behavior in parallel to progressive hippocampal antibody binding and a decrease of total and synaptic NMDARs, without affecting PSD95 or AMPAR. These effects gradually improved after stopping the antibody infusion, with reversibility of symptoms accompanied by restoration of NMDAR levels, establishing the pathogenicity of the antibodies (233). A need to develop new therapies targeting this direct pathogenic effect of autoantibodies derives from the fact that some patients suffer from severe symptoms and prolonged intensive care treatment in spite of aggressive immunosuppressive therapy. In these, long-lasting and therapy-refractive local autoantibody production in the CNS by plasma cells hidden within the BBB might be the cause (224). Future therapies could (1) interfere with binding of NMDAR-antibodies to its target antigen as has been successfully shown *in vivo* with the co-administration of Ephrin-B2 in an animal model (234); (2) apply BBB-permeable decoy approaches reducing effective NMDAR-antibody burden in the CNS; and (3) counteract symptoms *via* modulating antagonistic neurotransmitters to rebalance disturbed network functions.

### Induction of Structural Changes and Alterations in Signaling by Autoantibodies in Pemphigus Diseases

#### Epidemiology, Clinical Presentation, Histology, and Immunohistology of Pemphigus

Pemphigus diseases are a group of rare, but prototypical autoimmune blistering, skin conditions with autoantibodies against defined structural antigens of epidermal keratinocytes (235). There are two major types of pemphigus diseases, pemphigus vulgaris (PV) and pemphigus foliaceus (PF). The incidence depends on the population and is 1.6 per 100,000 adults in the Jerusalem area (236), but 0.7 for the total UK population (237) in PV. For PF, there is an endemic form (i.e., fogo selvagem) in Brazil, accounting for an incidence of 1–4 cases per 1,200 person years (238). Clinically, in PV, oral mucous membranes are usually affected, with facultative involvement of the skin (235). Affections in PF are only seen on the skin and blisters are even more fragile. Here, patients usually present with scaly crusted lesions, because blisters break very early after formation (235).

Histologically, pemphigus lesions show loss of intercellular adhesion of epidermal keratinocytes (239). All patients show intercellular staining of IgG within a biopsy of perilesional epidermis, whereas more than 80% of patients feature circulating IgG directed against keratinocyte surfaces in their serum (240). Multiple lines of evidence exist for the finding, that the main antigens targeted in pemphigus are desmogleins (Dsgs), desmosomal transmembrane glycoproteins that mediate epidermal cell–cell adhesion, and different expression (and compensation) of Dsg3 and Dsg1 within epidermis and mucous membranes explains the localization of blisters in PV and PF patients (235, 240, 241). Other antigens targeted in a minority of pemphigus patients may include other desmosomal cadherins (e.g., desmocollins, Dscs), classical cadherins or even other autoantigens (242–248). Knowledge of these antigens targeted in pemphigus patients allowed for development of serological tests as immunofluorescence and ELISA, and titers measured usually correlate with disease activity (see below) (249–251).

#### Brief Overview on Standard Treatment Options in Pemphigus

Standard present treatment is reviewed extensively elsewhere (235, 252), but critically depends on immunosuppression and modulation. Corticosteroids are effective within days when given systemically, likely because of increased synthesis of targeted Dsgs by keratinocytes, counteracting the desmosome-depleting effects of anti-Dsg antibodies in patients (253). Anti-CD20 therapy with rituximab represents potentially very effective therapy, leading to complete remission off all therapy in about 90% of pemphigus patients treated with one or more cycles of intravenous CD20+ B-cell-depleting rituximab (109). All of these options have their own limitations and challenges, and there are patients resistant to multiple regimens of therapy illustrating the need for a better understanding of the pathophysiology of pemphigus on a molecular level -including signaling pathways implicated into autoantibody-induced blistering- hopefully resulting in more specific therapy.

#### Autoantibodies Alone Cause Skin Pathology in Pemphigus

**Figure 8:**
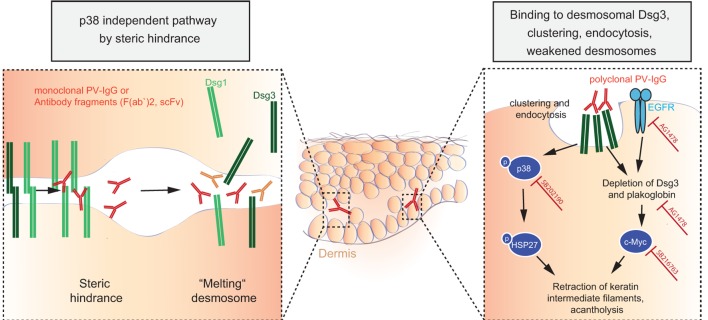
**Autoantibody-induced loss of cell–cell adhesion in pemphigus**. For explanation, please refer to the text.

Early experiments using passive transfer of human IgG from pemphigus patients into mice demonstrated dose-dependent recapitulation of human pathology (254). Disease induction is possible with bivalent F(ab′)2 and monovalent Fab′ fragments purified from PF patients’ sera as well with monovalent scFv fragments cloned by antibody phage display from pemphigus patients (255–258). Consequently, Fc-dependent mechanisms are dispensable in pemphigus pathophysiology, and autoantibodies directly mediate disease by interfering with the interaction of desmoglein molecules on the outside of keratinocytes, either by interference with homophilic trans- or cis-interaction of Dsg molecules (Figure 8) (259–261). Further evidence for direct interference with adhesion comes from epitope-mapping studies of pemphigus autoantibodies on domain-swapped and point-mutated Dsg1/Dsg3 molecules, mapping most of the dominant epitopes bound by patients’ sera to the aminoterminal ectodomains of Dsg1/3, which are critical for adhesion (262). Finally, pemphigus antibodies bind to calcium-dependent conformational epitopes, something seen in adhesion mediated by cadherins as well, again suggesting that patients’ autoantibodies do bind to domains important for adhesion. Interestingly, Dsg3-specific T cells have also been demonstrated to cause cutaneous pathology by inducing interface dermatitis when injected into lymphocyte-deficient mice (263).

#### Clustering, Internalization, and Depletion of Desmosomal Dsgs by Autoantibodies

Various researchers have been proposing other models, which are most likely not opposing the model of steric hindrance but rather complementing observed pathologies in pemphigus: the Dsg non-assembly depletion hypothesis describes how divalent anti-Dsg antibodies cross-link and cluster Dsgs, leading not only to internalization of non-junctional membrane-bound Dsgs but also resulting in prevention of incorporation of newly synthesized Dsgs into forming desmosomes (264, 265). Consequently, desmosomes become depleted of Dsgs and fail to provide adhesion. This model is supported by data from patients and from cell culture experiments, showing clustering of Dsg3/1 by Dsg3/1-specific autoantibodies (266–268), and by the observation that forced expression of Dsg3 by adenoviral delivery can prevent autoantibody-mediated Dsg3 depletion of desmosomes and acantholysis (267).

#### Autoantibody-Mediated Changes in Cell Signaling Contribute to Loss of Cell Adhesion in Pemphigus

The observations that polyclonal IgG from PV patients causes retraction of keratin intermediate filaments (thus contributing to intercellular loss of adhesion) in cultured murine wild-type keratinocytes and that plakoglobin (PG)-deficient mice do not show this reaction pointed to intracellular signaling mechanisms contributing to acantholysis (269). Further research then demonstrated that PG is a suppressor of c-Myc expression and that PV autoantibodies trigger c-Myc upregulation by depletion of PG together with Dsg3; increased c-Myc then leads to cell proliferation and weakened intercellular adhesion. These findings were corroborated by pharmacological inhibition of c-Myc, resulting in an inhibition of the ability of PV autoantibodies to cause acantholysis in mice (270, 271). As another signaling cascade extensively studied in pemphigus pathophysiology is the p38MAPK-signaling pathway (272–274). HSP27 and p38MAPK have been shown to be phosphorylated upon incubation of human keratinocyte cell cultures with PV-IgG (272, 273) and to be linked to internalization of Dsg3 (275). Blister formation was blocked *in vivo* by pharmacological inhibition of p38MAPK and its downstream targets when studied in the passive transfer mouse model for PV and PF (274, 276, 277), and this signaling cascade was also shown to be functional in patients’ skin (278). Downstream of p38, epidermal growth factor receptor (EGFR) signaling was shown to be activated in human keratinocytes after addition of PV autoantibodies, and again, inhibition of EGFR signaling prevented blistering induced by PV-IgG in mice (118). What has not been fully resolved is the issue of whether p38 activation is a primary event causing acantholysis or is secondary to initial loss of cell adhesion (235).

Attempting to integrate published, but sometimes seemingly contradictory data, recent research has pointed out that (i) steric hindrance and (ii) Dsg3 clustering, depletion, and signaling are distinct events. Monoclonal pathogenic antibodies can cause loss of intercellular adhesion through steric hindrance without relying on p38MAPK signaling, whereas polyclonal PV-IgG autoantibodies cause Dsg3 clustering and endocytosis *via* a p38MAPK-dependent pathway (235, 260). Recent data support these findings and suggested that, after induction of acantholysis and activation of p38MAPK signaling by polyclonal PV-IgG, effective modulation of the signaling pathways involved in acantholysis may even outbalance direct inhibition of Dsg3 binding by PV-IgG and rescue cell adhesion (279), potentially by upregulating other cell adhesion molecules not targeted by anti-Dsg3 antibodies (e.g., Dsg1, Dscs, cadherins).

#### Emerging Treatments and Novel Therapeutic Targets Based on Modulation of Signaling and Cell–Cell Adhesion

All these cell biological studies of signaling and desmosomal adhesion in pemphigus have led to innovative and new approaches to treatment. For example, cross-linking the adhesive interfaces of Dsgs by use of a specific tandem peptide successfully stabilized adhesion and inhibited PV-IgG-mediated activation of the central p38MAPK pathway and skin blistering (119). Overexpression of plakophilin-1, an intracellular armadillo protein that links desmosomal cadherins to keratin intermediate filaments of keratinocytes, resulted in hyperadhesive desmosomes that were significantly less prone to PV-IgG-mediated pathology (280). Introducing a point mutation into DP (DP-S2849G) led to inhibition of both Dsg3 depletion from the cell surface and keratin filament retraction caused by PV-IgG (281), by preventing protein kinase C-dependent phosphorylation of DP at that specific site. Since the protein kinase C inhibitor Bim-X has the same inhibiting effects, this compound may serve as an important new pharmacological tool in pemphigus.

Interestingly, studying the precise mechanisms of seemingly “boring” but very effective corticosteroid therapy in pemphigus disease can yield new therapeutic insights as well. As previously assumed, corticosteroids do upregulate Dsg3 transcription in primary human keratinocytes. In addition, recent data point to inhibition of Stat3 as a key mechanism. Similarly, inhibition of mTOR by rapamycin (i.e., sirolimus) is Stat3-dependent and upregulating Dsg3 transcription, explaining how PV-IgG-mediated effects are antagonized by rapamycin and corticosteroids (282).

Very recently, researchers in the pemphigus field have made an important contribution to the treatment of all autoimmune disease: engineered human T cells that express a chimeric autoantibody receptor (CAAR), consisting of the PV autoantigen, desmoglein (Dsg) 3, fused to CD137–CD3z signaling domains were generated. These Dsg3 CAAR-T cells showed specific cytotoxicity against cells expressing anti-Dsg3 BCRs *in vitro* and expand, persist, and specifically eliminate Dsg3-specific B cells *in vivo*. Thus, CAAR-T cells may provide an effective and also common strategy for specific targeting of autoreactive B cells in antibody-mediated autoimmune disease (117).

Taken together, these data on autoantibody-mediated changes of cell signaling in a group of prototypic autoimmune diseases illustrate that studying the potentially multifaceted roles of the target antigens is of the utmost importance: as shown for the desmosomal autoantigens in pemphigus diseases, Dsgs are not just structural components critical for adhesion but also critical regulators of signal transduction, affecting differentiation, cell homeostasis, and carcinogenesis (283–285). Integrating all these and dissecting the precise mechanisms of standard therapy may suggest promising new targets for therapy and modulation of autoimmunity.

## Autoantibody-Induced TTP: Anti-ADAMTS13 Antibodies

### Clinical Presentation, Epidemiology, Treatment, and Treatment Challenges

**Figure 9:**
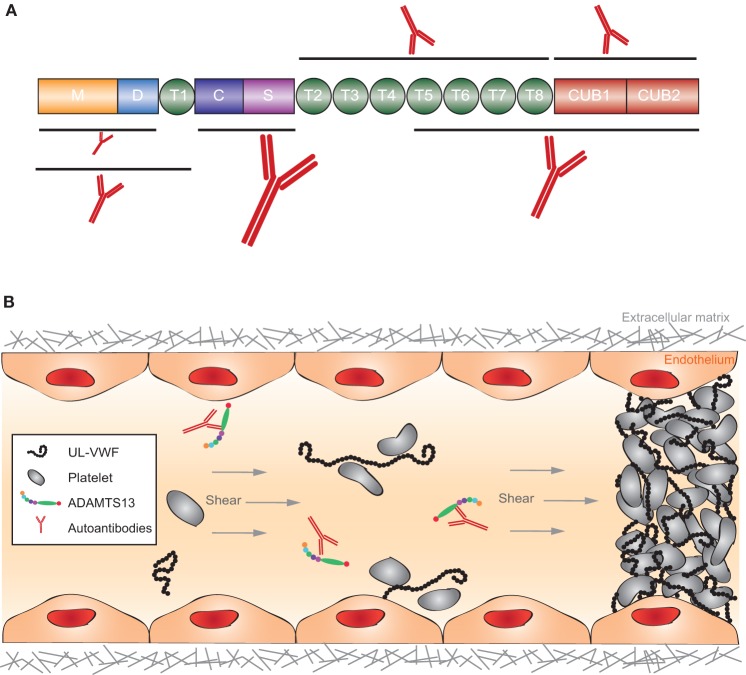
**Structure of ADAMTS13 and the frequency of autoantibodies targeting specific domains and graphical representation of the pathophysiology of acquired thrombotic thrombocytopenic purpura (TTP)**. **(A)** ADAMTS13 consists of a metalloprotease (M; orange) and disintegrin-like (D; blue) domain, a thrombospondin type-1 repeat (T1; green), a cysteine-rich (C; purple) and spacer (S; pink) domain, seven additional thrombospondin type-1 repeats (T2–T8; green), and two CUB domains (CUB1-2; red). Epitope mapping of anti-ADAMTS13 autoantibodies revealed that most patients (90–100%) have autoantibodies against the cysteine-spacer domain (indicated with the largest antibody). The size of the other antibodies in this figure demonstrates the relative frequency of these autoantibodies in the plasma of acquired TTP patients. **(B)** In normal conditions, UL-VWF released from activated endothelial cells is directly proteolysed by ADAMTS13, preventing spontaneous platelet binding. In the pathophysiology of acquired TTP, autoantibodies inhibit ADAMTS13 activity. UL-VWF multimers accumulate in the circulation and spontaneously bind platelets. This process results in the formation of VWF-rich microthrombi blocking the circulation in microcapillaries and arterioles.

Thrombotic thrombocytopenic purpura is a thrombotic microangiopathic disorder caused by a deficiency in the multidomain metalloprotease ADAMTS13 (A Disintegrin And Metalloprotease with ThromboSpondin type 1 repeats, number 13) (286, 287) (Figure 9A). In the absence of ADAMTS13, ultra-large hyperactive von Willebrand factor (VWF) multimers accumulate in the circulation and spontaneously bind platelets. The resulting VWF-rich microthrombi block capillaries and arterioles in different organs, thereby preventing oxygen supply (Figure 9B). Patients suffer from recurrent episodes of severe organ failure, which without urgent treatment can be fatal. Organ involvement is variable and can comprise multiple organs, most predominantly brain, heart, and kidney (287). TTP is clinically diagnosed by the occurrence of severe thrombocytopenia and hemolytic anemia (288, 289). It is assumed that platelets are consumed in the microthrombi and that red blood cells rupture as they are pushed through the blocked microcapillaries and arterioles resulting in the appearance of schistocytes, as well as increased levels of hemoglobin and haptoglobin. Tissue damage resulting from occlusive VWF-rich microthrombi furthermore results in increased levels of lactate dehydrogenase. TTP diagnosis is ascertained by determination of ADAMTS13 activity (<10%) allowing differentiating TTP from other microangiopathic disorders like hemolytic uremic syndrome (286). Congenital TTP is caused by the presence of mutations in the ADAMTS13 gene and occurs in ~5% of the TTP patients. About 95% of TTP patients suffer from acquired TTP. These patients develop anti-ADAMTS13 autoantibodies and hence suffer from the autoimmune form of the disease (290). Acquired TTP with severe ADAMTS13 deficiency is an orphan disease with an incidence rate of around 2–6/1,000,000 patients per year (286, 291). Since in a subset of TTP patients in remission, ADAMTS13 activity is still below 10%, secondary triggers like pregnancy, infection, and surgery have been suggested to induce TTP.

Infusion with fresh frozen plasma is the treatment of choice for congenital TTP patients to provide sufficient levels of functional ADAMTS13. Plasma exchange, where patient plasma is exchanged with fresh frozen plasma, is used to treat acquired TTP patients in order to remove anti-ADAMTS13 autoantibodies and to supply active ADAMTS13. To treat the underlying autoimmune disorder in acquired TTP, an immunosuppressive corticosteroid therapy is started together with the plasma exchange therapy (288, 289, 292). Rituximab, the anti-CD20 mAb targeting mature and memory B-cells, is now additionally administered to those acquired TTP patients experiencing a suboptimal response to plasma exchange and corticosteroid therapy and to patients suffering from TTP relapses (293–295). Although standard therapy (plasma exchange and immunosuppressive therapy) reduces the mortality rate in 80–90% of TTP patients, mortality remains at 10–20% (292). In addition, plasma exchange is a challenging therapy for TTP patients as the patients have a poor clinical condition and a high number of plasma exchanges is required with large volumes of plasma. Major complications linked with plasma exchange are observed and are related to systemic infection, venous catheter obstruction, hypotension, hypoxia, venous thrombosis, non-fatal cardiac arrest, and anaphylaxis (288).

### Pathogenesis of Acquired TTP

Autoantibodies play a predominant role in the pathophysiology of acquired TTP. Anti-ADAMTS13 autoantibodies either inhibit ADAMTS13 function and/or clear ADAMTS13 from the circulation (296). Both processes result in the absence of active ADAMTS13 in patient plasma leading to the accumulation of prothrombotic ultra-large VWF multimers and spontaneous VWF-rich microthrombi formation, the hallmark of acute TTP episodes. The presence of both, free anti-ADAMTS13 autoantibodies and circulating autoantibody–antigen immune complexes, has been described in plasma of acquired TTP patients (297). Anti-ADAMTS13 autoantibodies are mainly of the IgG class, with IgG4 being the most prevalent, but IgM and IgA autoantibodies have also been reported (298). The IgG4 subclass is also most predominantly observed in circulating immune complexes (297). Accordingly, moderately elevated complement activation is observed during acute TTP episodes (299–301). It is however unclear whether the circulating autoantibody–ADAMTS13 immune complexes activate complement and play a role in the pathophysiology of TTP.

Antibody titers change during acute phases and remission. The autoimmune response in acquired TTP is polyclonal but with immunodominant epitopes in the ADAMTS13 spacer domain (Figure 9A) (290, 302). Plasma screening of acquired TTP patients against the different ADAMTS13 domains revealed that 90–100% of acquired TTP patients have autoantibodies with an epitope in the spacer domain (Figure 9A) while 30–50% of the patients additionally have antibodies directed against other ADAMTS13 domains (M, D, T, C, T2-8, and CUB1-2, Figure 9A) (290, 296, 302). Many anti-spacer autoantibodies have been cloned from acquired TTP patients and were shown to have a strong, weak, or absent *in vitro* inhibitory effect on ADAMTS13 function (296, 302–305). The ADAMTS13 spacer domain contains a major exosite for binding to its substrate VWF explaining why anti-spacer domain autoantibodies can inhibit ADAMTS13 function. Non-inhibitory cloned anti-spacer antibodies are expected to clear ADAMTS13 from the circulation as it was demonstrated by Thomas et al. that the IgG fraction isolated from acquired TTP patients contains non-inhibitory anti-ADAMTS13 autoantibodies and that ADAMTS13 antigen levels were low in these patients (296). Only a few anti-ADAMTS13 autoantibodies with an epitope outside the spacer domain have been cloned. These comprise autoantibodies with an epitope in the ADAMTS13 metalloprotease, disintegrin, and CUB domains, but their effect on ADAMTS13 function has not yet been studied (305, 306). Anti-metalloprotease autoantibodies could inhibit ADAMTS13 function, as the metalloprotease domain harbors the active site of the enzyme. Anti-disintegrin or anti-CUB autoantibodies could have similar effects, as these domains are involved in binding and docking ADAMTS13 to VWF, respectively. However, in addition, autoantibodies could merely clear ADAMTS13 from the circulation. Whether deposition of circulating immune complexes in tissues also play a role in the pathophysiology of acquired TTP remains to be determined.

We showed that injection of a murine anti-metalloprotease mAb that potently inhibited ADAMTS13 function *in vitro* could induce acquired TTP in baboons (307). This experiment shows that anti-ADAMTS13 antibodies play a central role in the pathophysiology of acquired TTP. Recently, it was shown that injection of a cloned inhibitory anti-spacer single chain (sc) Fv autoantibody into mice also resulted in acquired TTP when TTP symptoms were triggered with Shiga toxin or recombinant VWF (305, 308). TTP symptoms presented due to full inhibition of ADAMTS13 activity in both animal models, as animals injected with non-inhibitory control antibodies did not develop TTP symptoms. However, not all acquired TTP patients have strong inhibitory anti-spacer autoantibodies and 30–50% of the patients also have autoantibodies against other ADAMTS13 domains.

Hence, animal models where individual autoantibodies or mixtures of these autoantibodies are injected need to be performed to further unravel the contribution of all these autoantibodies in the pathophysiology of acquired TTP. Indeed, since the immune response in acquired TTP is polyclonal, it is reasonable to speculate that multiple autoantibodies will contribute to inhibition and/or clearance of ADAMTS13 in 30–50% of the patients. It is currently less obvious to study the clearance of anti-ADAMTS13 autoantibodies as most available cloned autoantibodies are either mounted on an IgG1 or IgG4 scaffold, or are scFv fragments. Mouse models cannot be used to perform these studies as clearance mechanisms in mice are too different from those in humans.

The mechanism of autoantibody development in acquired TTP patients is currently not known but both environmental and genetic factors will probably play a role as shown in other immune diseases. Indeed, HLA-DRB1*11 has been shown to be overrepresented in patients with acquired TTP compared to controls (309). The role of specific CD4+ T-cells in acquired TTP has only recently been investigated. It was shown that ADAMTS13 is endocytosed by dendritic cells *via* the macrophage mannose receptor (310). Peptides derived from the carboxy-terminal CUB2 domain of ADAMTS13 were preferentially presented, showing that the CUB2 domain contains potential immunodominant T cell epitopes (310). Investigating ADAMTS13 reactive CD4+ T cells from acquired TTP patients showed that CUB2 domain reactive CD4+ T cells might be involved in the etiology of acquired TTP (311).

### Emerging Treatments and Novel Therapeutic Targets

Recombinant (r)ADAMTS13 is currently under development as an alternative treatment for TTP. It is effective to treat congenital TTP symptoms in mice (312) and acquired TTP symptoms in rats, where rADAMTS13 was able to overcome circulating inhibitors and reconstitute ADAMTS13 activity (313). A Phase I clinical study is currently going on to assess rADAMTS13 in the treatment and prophylaxis of congenital TTP (NCT02216084). Apart from providing active ADAMTS13 to patients, a treatment has been developed that prevents the formation of VWF-rich microthrombi, which play a detrimental role in TTP pathophysiology. Others and we showed that administration of an antibody blocking the binding between VWF and platelets results in the prevention and treatment of acquired TTP in preclinical animal models (314, 315). The inhibitory anti-VWF nanobody caplacizumab in combination with standard therapy was tested in clinical trials and induced a faster resolution of acute TTP episodes compared to placebo in acquired TTP patients (112). A second therapy that aims at preventing or reducing VWF–platelet interactions is the FDA approved drug *N*-acetylcysteine (NAC) currently used to treat acetaminophen overdose or to decrease the viscosity of mucous secretions in respiratory disorders. NAC reduces disulfide bonds in mucins and was shown to also reduce disulfide bonds in VWF multimers, thereby reducing the formation of VWF-platelet thrombi in mesenteric venules of mice (316). NAC has been administered to patients with refractory TTP but the outcome has been variable (317–320). To determine the clinical value of NAC, patients are currently being recruited (ClinicalTrials.gov: NCT1808521). Another drug that is evaluated to treat refractory TTP is the proteasome inhibitor bortezomib. Although case reports have suggested a benefit for the treatment of refractory TTP (73, 320), possibly by eliminating autoreactive plasma cells producing anti-ADAMTS13 antibodies (321), prospective clinical trials are needed to investigate the effectiveness of this drug in the treatment of refractory TTP.

## Autoantibody-Induced Cell Lysis: Idiopathic Thrombocytopenic Purpura (ITP) and Autoimmune Hemolytic Anemia

Immune thrombocytopenic purpura (formerlyITP) and autoimmune hemolytic anemia (AIHA) are autoimmune diseases that are characterized by reduced levels of erythrocytes or platelets resulting in anemia or platelet deficiency, respectively. AIHA and ITP may both appear as secondary disease in consequence of a different medical condition or can be induced by medical treatments. In contrast to these secondary forms, the pathogenesis of the primary diseases is not fully understood but in the majority of patients, autoantibodies directed against erythrocytes or platelets, respectively, can be detected.

Primary AIHA seems to be exclusively caused by such autoantibodies while in primary ITP these can only be detected in 60% of the patients (322). In the residual cases, a contribution of cytotoxic T cells is considered (323) but it is likely that autoantibody levels are simply undetectable due to the quantitative binding to platelets and their subsequent fast elimination. The removal and destruction of erythrocytes or platelets in AIHA or ITP, respectively, is mainly mediated by Fcγ-receptor (FcγR)-based mechanisms in cases where the autoantibodies are of the IgG class. While the role of complement in the removal of platelets does not play a prominent role in ITP (324), the situation in AIHA is somewhat different. In a considerable fraction of patients, IgM autoantibodies are involved that efficiently can activate complement, which is not the case when erythrocytes are opsonized by IgG (325). As the different pathways of red blood cell destruction require also different treatments, the determination of the involved autoantibody Ig class is essential.

The contribution of FcγR-mediated effector functions to the pathogenesis of ITP triggered by opsonized platelets has identified an association with reduced expression levels or impaired signaling of inhibitory FcγRIIB (326, 327) as well as the presence of a functional copy of the activating FcγRIIC gene (328). Similar genetic association studies have been conducted for the role of the activating FcγRs. For the FcγRIIA H/R131 dimorphism, an elevated prevalence of the FcγRIIA-R131 allele, which has a lower affinity for IgG2 and a higher one for IgG3, was observed (329, 330), while for that of FcγRIIIA (F/V158) diverging results have been reported in ITP patients (331–333).

While the impact of activating FcγRs on both human heterogeneous diseases remains unclear, studies in mouse models of experimental AIHA and ITP could demonstrate a contribution to disease severity mediated by activating FcγRs, namely, FcγRIIIA (329, 334–337). In a therapeutic approach, the hypothesis was tested if blockade of FcγRIII can improve ITP in chronic refractory patients. Using an anti-FcγRIIIA antibody, around 50% of the included patients responded with significantly improved platelet levels but treatment was accompanied by severe adverse events. Using several engineered versions of the anti-FcγRIII antibody, the authors demonstrated that the observed adverse events were independent of Fc-mediated effector functions or immunogenicity (338) but rather depended on the dimerization of FcγRIII by both Fab fragments of the used antibody. This finding was supported by a study using a passive ITP mouse model in which a monovalent anti-FcγRIII antibody was used. This construct which blocks immune complex-mediated engagement of FcγRIII mediated an efficient inhibition of antibody-dependent platelet removal without triggering adverse events (339).

In a related approach, a soluble FcγR was used with the intention to compete with cellular FcγRs for the binding of opsonized platelets. As a consequence, the platelet binding to FcγRs is blocked and destruction and cellular activation of the immune cells is inhibited (340). In chronic ITP patients, the drug was well tolerated and induced a sustained platelet response during the 3-month follow-up period (341). Both approaches underline the relevance of the FcγR system in autoimmune diseases and may provide new treatment options for the management of ITP, AIHA, and eventually other antibody-mediated autoimmune diseases.

## Autoantibody-Induced Vascular Inflammation

Antineutrophil cytoplasmic autoantibody (ANCA)-associated vasculitides (AAV) comprise a group of three different rare and potentially life-threatening chronic inflammatory vessel diseases of unknown etiology: granulomatosis with polyangiitis (GPA), microscopic polyangiitis (MPA), and eosinophilic granulomatosis with polyangiitis (EGPA). GPA is associated with proteinase 3 (PR3)-specific ANCA (PR3-ANCA), whereas MPA and—less commonly—EGPA are associated with myeloperoxidase (MPO)-specific ANCA (MPO-ANCA). Usually P33 and PR3-ANCA are detected by indirect fluorescence (see below). AAV predominantly affect small vessels, i.e., intraparenchymal small arteries, arterioles, capillaries, and venules. The vasculitis is histopathologically characterized by fibrinoid necrosis and paucity of immune complexes (“pauci-immune vasculitis”). In addition to systemic vasculitis, GPA displays a peculiar propensity for a predominantly extravascular necrotizing granulomatous inflammation mainly affecting the upper and/or lower respiratory tract. In EGPA, extravascular eosinophilic infiltration is found in various tissues. In contrast, MPA displays no extravascular granulomatous inflammation (342–344).

In central Europe, GPA is the most common form of AAV (incidence 10 per million, prevalence 98 per million), followed by MPA (incidence 2 per million, prevalence 28 per million) and EGPA (incidence 1 per million, prevalence 23 per million). However, these numbers may underestimate the true incidence and prevalence of AAV due to referral bias. The mean age at disease manifestation lies between 50 and 70 years. Women and men are equally affected (345). AAV as a group are characterized by diversity and at the same time considerable overlap of clinical and pathological features. Malaise, flu-like symptoms, arthralgia, and myalgia often herald the onset of AAV. Respiratory tract manifestations are the most frequent presenting features in GPA. Involvement of upper and/or lower airways with rhinitis, sinusitis, and pulmonary infiltrates is a distinctive feature of GPA. Asthma, pulmonary infiltrates, and hypereosinophilia with eosinophilic vasculitis and inflammation affecting various organs are characteristic features of EGPA. Pulmonary-renal syndrome with alveolar hemorrhage as a result of capillaritis and rapidly declining renal function due to focal segmental necrotizing glomerulonephritis with crescent formation affects the majority of patients with full-blown GPA and MPA. Renal involvement is less common in EGPA. Owing to its systemic nature, the vasculitis may affect any organ in AAV. Localized forms of GPA restricted to the upper and/or lower respiratory tract and a renal-limited form of MPA are less commonly encountered (343).

Prior to the introduction of immunosuppressive therapy, AAV used to be an inevitably fatal condition. With the advent of corticosteroids and establishment of immunosuppressive cytotoxic treatment protocols in randomized trials, the outcome and survival rates have considerably improved (343, 346). However, the mortality ratio continues to be increased (2.6, 95% CI 2.2–3.1) compared with an age- and sex-matched general population (347). Despite further advances in treatment such as the use of rituximab for the induction and maintenance of remission, the course of AAV remains characterized by chronicity, risk of relapse, and complicating comorbidities. Thus, major goals of research are the prevention of disease through a personalized medical approach allowing the evaluation of predisposing genetic risk factors for disease manifestation and relapse, the determination of triggering environmental factors, and the development of combined targeted therapies intervening early in the break of tolerance and with mechanisms fostering chronic non-resolving inflammation in AAV (346).

Antineutrophil cytoplasmic autoantibodies are highly sensitive and specific for AAV. Combining immunofluorescence technique and ELISA (see below) for the detection of ANCA yields a sensitivity of 70% and specificity of 99% for AAV (348). ANCA levels do not strongly reflect disease activity. Therefore, ANCAs cannot be used for the guidance of immunosuppressive therapy in clinical practice (349). However, persistence of ANCA after the induction of remission is strongly associated with an increased risk for relapse in AAV (350). ANCAs are rarely detected in healthy controls. Interestingly, there is no evident co-occurrence with other autoantibodies suggesting differences in the break of tolerance between different autoimmune diseases (63). ANCA with specificity for a subset of immunodominant epitopes are associated with disease activity in AAV (351). ANCAs induce respiratory burst and degranulation of cytokine- and complement factor C5a-primed neutrophils *in vitro* (352, 353). Prestimulation of neutrophils with cytokines promotes translocation of the target antigens of ANCA on the cellular surface. Interaction of ANCA with both its target antigen on the cellular membrane and Fcγ-receptors IIa or IIIb is required for the full activation and subsequent degranulation of neutrophils (354, 355). Binding of ANCA to both, the membrane-bound ANCA targets MPO and PR3 and Fcγ-receptors IIa or IIIb, generates activation signals distinct form conventional Ig-induced activation *via* Fcγ-receptors (356). Genes of pro-inflammatory mediators are upregulated following stimulation with ANCA (357). ANCAs facilitate adhesion and transmigration of cytokine-primed neutrophils across the endothelial cell barrier *in vitro* and *in vivo* as observed in flow-chamber experiments and by intravital microscopy (358, 359). Transfer of MPO-ANCA generated from MPO-knockout mice immunized with mouse MPO induces glomerulonephritis and vasculitis in susceptible mice. The genetic background of the mouse determines the severity of glomerulonephritis in these models (360). The alternative complement pathway is activated in AAV. Complement factor C5a receptor deficiency ameliorates MPO-ANCA-induced experimental glomerulonephritis in mice (361). Owing to structural and biologic differences between human PR3 and its rodent homolog, the development of models for PR3-ANCA-induced vasculitis has remained a challenge. Pathogenicity of PR3-ANCA resulting in acute vascular damage has been demonstrated in immunodeficient chimeric mice following injection of human hematopoietic stem cells and PR3-ANCA (362). However, extravascular necrotizing granulomatous inflammation was not found in the respiratory tract of these mice, suggesting that the pathogenesis of granulomatous inflammation may be separate from acute systemic vasculitis and in particular T-cell-dependent in GPA (362, 363).

Clinical observations, the efficacy of B-cell-depleting therapy, and evidence from *in vitro* and *in vivo* experiments taken together all lend support to the concept that ANCAs are pathogenic and induce a necrotizing small-vessel vasculitis. However, unresolved questions concern the cascade of events leading to the induction of pathogenic ANCA and the pathogenesis of extravascular granulomatosis in GPA and EGPA. Thus, key elements of the pathogenesis of AAV and its characteristic immune pathology remains to be elucidated (346). While the uptake of apoptotic cells and debris, a process known as efferocytosis, is usually immunologically silent in healthy individuals, upregulation of PR3 on the cell surface of apoptotic neutrophils interferes with normal efferocytosis in GPA. Increased PR3 expression on apoptotic neutrophils induces the secretion of inflammatory cytokines including granulocyte-colony stimulating factor (G-CSF) by macrophages *in vitro* and *in vivo*. In turn, G-CSF upregulates PR3 expression on maturing neutrophils from GPA patients in the bone marrow. Thus, a G-CSF-driven autoamplificatory loop sustains non-resolving inflammation in GPA. Moreover, PR3-expressiong apoptotic cells skew the cytokine response of plasamocytoid dendritic cells toward the generation of Th2 and Th9 cytokines. In the presence of PR3-ANCA, the cytokine response was further skewed toward a Th17 response in a murine model. Accordingly, skewing of the cytokines response with an increase in circulating Th2, Th9, and Th17 cells was also detected in GPA patients (364). Notably, ectopic lymphoid structures and plasma cells displaying signs of autoreactivity are found in granulomatous inflammation rich in neutrophils in GPA (365). Once tolerance is broken and pathological autoreactivity toward PR3 and MPO established in AAV, the autoreactive immune response is further self-amplified by neutrophil extracellular traps (NETs). NETs are released by ANCA-stimulated neutrophils and contain the targeted autoantigens PR3 and MPO (366).

Taken together, the abovementioned studies suggest an immunological paradox in which cell death, i.e., necrosis, is not only the endpoint of ANCA-induced neutrophil degranulation and inflammation in AAV but also at the same time represents the starting point of a cascade of pathophysiological events leading to chronic non-resolving extravascular and vascular inflammation and the breakdown in self-tolerance. This paradox, however, could explain why neutrophil-derived antigens become the target of pathological autoreactivity and inflammation is sustained and does not resolve in AAV. The cascade may initially be triggered by environmental factors, e.g., infections, in genetically susceptible persons. G-CSF and/or IL-17 could become new therapeutic targets for the treatment of AAV. It is inferred that targeting G-CSF and IL-17 interferes with fundamental pathophysiologic mechanisms driving non-resolving chronic inflammation and the break of tolerance in AAV.

## Autoantibody-Induced Inflammation

### Autoantibody-Induced Cutaneous Inflammation: Pemphigoid Diseases (PD)

Pemphigoid diseases are characterized and caused by autoantibodies against distinct structural components of the dermal–epidermal junction. Junction proteins link the cytoskeleton of the basal keratinocytes to the extracellular matrix of the dermis. Binding of PD autoantibodies leads to the separation of the epidermis and dermis by a complex, yet relatively well-understood process. PDs comprise eight distinct disorders for which the molecular target antigens have been identified (367). Of these, due to the availability of well-defined animal models, bullous pemphigoid [BP, autoimmunity against type XVII collagen (COL17, BP180)] and epidermolysis bullosa acquisita [EBA, autoimmunity against type VII collagen (COL7)] are particularity well studied (368–371). We here will focus on EBA because the expression of COL7 beyond the skin, i.e., in the gastrointestinal tract (372, 373), leads to a severe and difficult-to-treat clinical presentation. The cutaneous manifestations in EBA are heterogeneous: two major clinical subtypes have been described. The mechanobullous (non-inflammatory, classical) variant of EBA is characterized by skin fragility, tense blisters, scaring, and milia formation preferably localized to trauma-prone sites. The inflammatory variant mimics other PDs, and widespread vesiculobullous eruptions are present. Independent of the clinical variation, extracutaneous manifestations are frequently observed. These include ocular, oral mucosa, esophagus, anal, vaginal, tracheal, and laryngeal lesions, which can lead to blindness, esophageal strictures, hoarseness, impaired phonation, and may led to irreversible respiratory distress (374). Overall, EBA is notoriously difficult to treat, and often long-term use of high doses of corticosteroids in combination with other immunosuppressants is required to achieve clinical remission (375, 376). Thus, there is a high, and so far unmet medical need, for the development of novel treatment strategies for patients with EBA.

**Figure 10:**
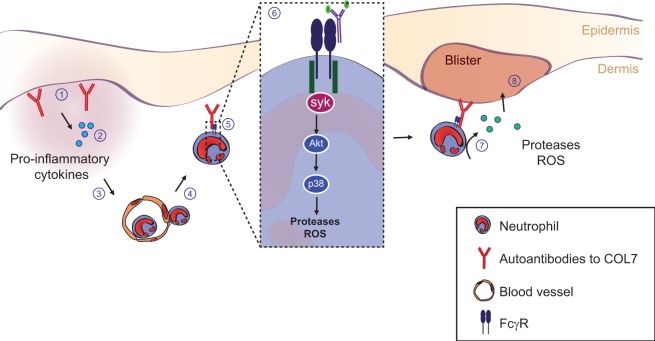
**Pathogenesis of inflammation and blistering in the pemphigoid disease epidermolysis bullosa acquisita**. Details are explained in the text, where the numbering corresponds to the labeling used here.

One of the breakthrough discoveries in EBA was the identification of COL7 as the autoantigens in the late 1980 (377). Based on this insight, the pathogenic relevance of COL7 autoantibodies has been demonstrated (i) *in vitro* by demonstrating digestion of the dermal–epidermal junction in skin specimen incubated with anti-COL7 IgG or IgA and neutrophils (378, 379), (ii) *in vivo* by induction of inflammation and blistering in mice by transfer of anti-COL7 IgG (380, 381) or by immunization (382, 383), and (iii) clinically by the observation of a correlation of circulating autoantibody titers with clinical disease severity (384, 385). Subsequently, use of these animal models has greatly contributed to our current understanding of EBA pathogenesis. The pathogenesis of blistering and inflammation in EBA can be divided in the following steps (Figure 10).

(1) The pathology-triggering event in EBA is without doubt the binding of the autoantibodies directed against COL7.
(2) This binding triggers the generation and/or the release of pro- and anti-inflammatory mediators, such as cytokines and complement. Of these, C5a, GM-CSF, CXCL1/2, TNF, and leukotriene B4 promote inflammation (44, 380, 386–389), while IL-1ra and IL-6 have profound anti-inflammatory activities (390, 391). These insights have almost exclusively been obtained from EBA mouse models, where inhibition of C5a, GM-CSF, CXCL1/2, or TNF impaired disease induction and/or ameliorated or even improved already manifest EBA. Conversely, blockade of IL-6 led to a deteriorating clinical EBA phenotype, while treatment with recombinant IL-6 or anakinra had beneficial effects (371). So far, no data on the source of these mediators have been published. Recent data, however, suggest that the diversity of the cutaneous microbiome has a significant impact on the expression of inflammatory mediators in the skin (392): in immunization-induced experimental EBA, the clinical outcome, i.e., development of clinically manifest disease, was associated with the diversity of the cutaneous microbiome. Mice with a relatively low diversity of cutaneous microbiota developed clinical EBA, while mice with a relatively high diversity were protected from disease induction. Of note, low diversity of cutaneous microbiota was also associated with higher cutaneous expression of TNF and CXCL1, as well as CD11c. This indicates that skin-resident cells or keratinocytes may be a source of these cytokines known to modulate blistering and inflammation in EBA. In addition, excess production of IL-10, i.e., induced by polyclonal B cell activation, has been shown do directly dampen C5a-driven inflammatory responses in EBA (56).
(3) Overall, this mediator release prompts a vascular response, characterized by an increased expression of endothelial adhesion molecules in cutaneous vessels (393), which allows the CD18-dependent extravasation of Gr-1+ myeloid cells into the skin (394).
(4) Within the skin, myeloid cells attach to the skin-bound immune complexes *via* specific Fc gamma receptors (395, 396). Regarding the activating Fc gamma receptors, the Fc gamma receptors IIA and IIIB mediate blistering in man, and the Fc gamma receptor IV in the mouse. At least in the mouse, the inhibitory Fc gamma receptor IIB has protective effects. These findings identified activating Fc gamma receptors as a potential drug target in EBA and other PDs. Indeed, if the binding of activating Fc gamma receptors to the skin-bound immune complexes is blocked, i.e., by removal of the sugar residues as Asn297 (397, 398), or by treatment with recombinant CD32-Fc (399), the induction of blistering can be prevented or even improved in mice with experimental EBA. Furthermore, the inhibitory Fc gamma receptor IIB is required to mediate the protective effect of high doses IgG in this model (400, 401).
(5) The Fc gamma receptor-mediated engagement of the myeloid effector cells to the tissue-bound immune complexes triggers a signaling process that initially triggers Syk and Src family kinases (115, 402). Downstream signaling involves PI3K beta (403), PDE4 (120), as well as RORα (391). These insights into the pathogenesis of EBA have led to the identification of several novel therapeutic targets for the treatment of the disease. For example, pharmacological inhibition of all of the above pathways hinders disease induction, and in some cases even improves already established disease when therapeutically applied.
(6) Ultimately, this process leads to the release of ROS *via* activation of the NADPH oxidase (394) and proteases (404), which cause blistering. For example, mice deficient for NCF1, a subunit of the neutrophil NADPH oxidase, are completely protected from induction of antibody transfer-induced EBA (394). Regarding proteases, blockade of neutrophil elastase or gelaninase B almost completely abolished dermal–epidermal separation of cryosections of skin incubated with EBA patient sera and leukocytes (404). In line, neutrophil elastase deficient mice are completely protected from blister induction by transfer of anti-type XVII collagen antibodies (405). Recent data also suggest that T cells can modulate this myeloid-driven inflammation. More specifically, γδ and NK-T cells promote inflammation and blistering by enhancing the migratory capabilities of myeloid cells (406).
(7) Quite recently, the actin remodeling protein Flightless I (Flii), known to be involved in would healing (407), has been identified as a potential therapeutic target for the treatment of EBA. In accordance to observations in would healing, overexpression of Flii led to a more severe clinical phenotype in antibody transfer-induced EBA (408), while pharmacological inhibition had therapeutic effects in mice with EBA (409). These findings indicate that processes controlling would healing are important to resolve inflammation and blistering, opening new avenues in our understanding of EBA pathogenesis, as well as pointing to so far neglected potential therapeutic targets.

Based on this detailed understanding of autoantibody-induced pathology in PD, several therapeutic targets have emerged. In my personal opinion, topical treatment using small molecules inhibiting signal transduction will have a significant impact on the management on patient care.

### Autoantibody-Induced Carditis

Myocarditis, defined as an inflammation of the myocardium, is a major cause of heart failure in young adults. Furthermore, myocarditis can lead to dilated cardiomyopathy (DCM), being the most frequent cause for heart transplantation (410). DCM is characterized by progressive depression of myocardial contractile function and ventricular dilation. Clinical manifestation of myocarditis ranges from patients without any relevant symptoms to acute cardiogenic shock. Most patients display flu-like symptoms, palpitations, or arrhythmias. Myocarditis is diagnosed by histological examination of endomyocardial biopsies (EMB). In echocardiography, CT and MRI an impaired wall movement and reduced ejection fraction might be observed. Changes in electrocardiogram can include ST elevation, heart block, and low voltage. Elevated levels of troponin T (TnT) and troponin I (TnI) suggest unspecific myocyte damage (410–412). Apart from infectious agents, like virus or bacteria, myocarditis can be caused by autoimmune reactions. Such an autoimmune myocarditis is characterized as histological confirmed myocarditis with no detectable viral genome in EMB (413) and detection of autoantibodies.

**Figure 11:**
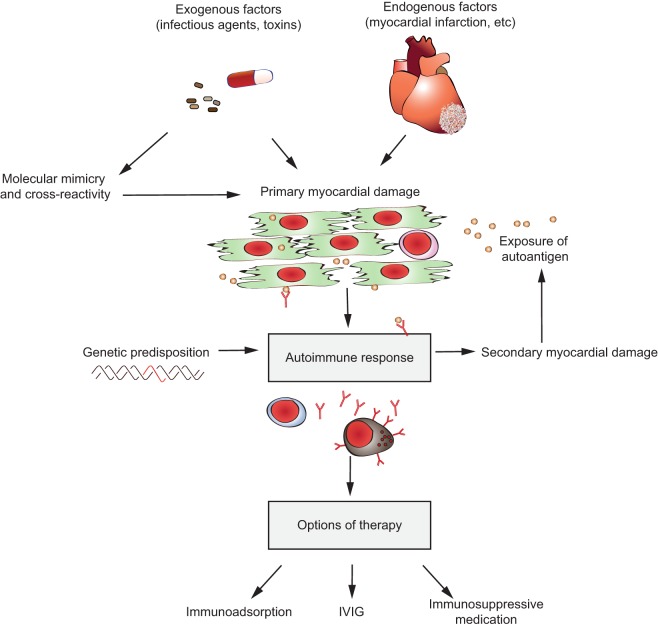
**Potential pathomechanisms in autoantibody-induced carditis and therapeutic options**. Release of self-antigens through cardiomyocte damage caused by exogenous or endogenous factors can induce an autoimmune response against myocardial tissue in genetic predisposed patients leading to secondary myocardial damage. Therapeutic options could be immunoadsorption, treatment with intravenous immunoglobulin (IVIG) and/or immunosuppressive medication.

The induction of an autoimmune response against the heart can be a consequence of cardiac injury induced by endogenous or exogenous factors (Figure 11). Triggering agents could be acute infections and toxic or ischemic events resulting in presentation of potentially antigenic determinants to the immune systems. Furthermore, molecular mimicry and cross-reactivity may play an important role. The resulting autoimmune reaction could lead to perpetuation of immune-mediated cardiac damage involving either cellular (e.g., T-cell), and/or humoral (e.g., B-cell) immune responses. Co-activation of both the innate and the adaptive immune system is possible (414). Thus, autoantibodies could be observed in all forms of inflammatory cardiomyopathies (415). These autoantibodies are targeted against various self-antigens, some of which are specific for heart cells. Already in 1987, Neu and colleagues demonstrated the presence of heart-specific autoantibodies following murine Coxsackievirus B3 myocarditis (416). Furthermore, it has been reported that heart-reactive antibodies were present in 59% of patients with myocarditis when rat tissue was used as substrate, whereas 20% of patients with DCM and less than 5% of healthy controls displayed antibodies against rat heart tissue (417). A study by Caforio et al. showed a higher frequency of circulating autoantibodies in relatives of DCM patients, which might be a possible predisposition to develop DCM (418). Another possible genetic predisposition for developing an autoimmune myocarditis could be observed in animal models. Here, mice of different genetic background showed variation in susceptibility to develop an inflammation of the myocardium after viral infection or immunization with heart-specific autoantigenes (419, 420).

Negative inotropic effects can cause impairment of cardiac function through autoantibodies. Moreover, apoptosis of cardiomyocytes and activation of the complement system may play a role (414). In fact, autoantibodies against adenine-nucleotide translocase induce functional consequences, which could be shown *in vitro* (421).

The “Etiology, Titre-Course, and Survival Study” investigates the prevalence and kinetics of autoantibodies in different forms of heart failure in a large multicenter trial (422). Moreover, various studies have investigated the treatment of autoimmune myocarditis beyond regular heart failure medication. The use of anti-inflammatory drugs like Igs, corticosteroids, azathioprine, and cyclosporine is one therapeutic approach (423, 424). However, for an immunosuppressive therapy, patients have to be virus-negative, because patients with biopsy-based exclusion of virus respond much better to the therapy (425, 426). Another approach is the treatment with IVIG. Here, an improved recovery of left ventricular function with a better survival tendency during the first year could be observed (427). The mode of action of IVIG preparation may be based on the binding and neutralization of circulating autoantibodies (428). In addition to IVIG therapy, immunoadsorption (IA) can be also used to eliminate cardiotoxic antibodies in the patient’s plasma. Here, the antibodies bind to a column adsorbing IgG with higher affinity than IgA and IgM. At least 18 h after the last IA treatment, patients receive polyclonal IgG infusion to restore their IgG levels. Due to IA, functionally active cardiac autoantibodies are removed resulting in improved hemodynamic parameters and biopsy-proven decrease in lymphocytic infiltration and expression of cellular adhesion molecules (423). According to this, a study by Wallukat et al. showed that IA removed circulating IgG3 antibodies targeting β1-AR. The clearance of these antibodies led to an improvement of heart function and a shift to a lower NYHA state (429).

Despite various research approaches, the pathogenesis of autoimmune myocarditis is yet not fully understood. A better understanding of the mechanisms leading to an autoimmune response against cardiac antigens may contribute to a more specific therapy. To investigate the role of autoantibodies and their influence on disease progression, different mouse models, e.g., immunization with cardiac peptides derived from TnI or myosin, have been established (430).

Taken together, different studies demonstrate an association between autoimmune damage related to cardiovascular disorders and the generation of functional antibodies against various proteins. Some therapeutic approaches reached clinical relevance but further studies are needed to confirm their benefit.

### Autoantibody-Induced Joint Inflammation: RA

With an estimated incidence of 0.6–1%, RA represents a major challenge for an aging Western population (431). Despite the availability of highly efficient therapies, such as the blockade of pro-inflammatory cytokines, B cell depletion, or a more generalized immunosuppression, many patients do not respond to therapy or become refractory to treatment. Thus, an in-depth understanding of the underlying mechanisms causing joint inflammation and bone destruction may be critical to identify novel therapeutic avenues. Although the final proof of an essential role of autoantibodies in the disease pathology of RA in humans is lacking, the strong association of autoantibodies recognizing cyclic citrullinated proteins (CCP-specific antibodies), which were shown to directly impact osteoclast activity, or the presence of rheumatoid factor antibodies, makes this scenario highly likely (431). Moreover, certain activating FcγR alleles were shown to be associated with the incidence or severity of disease, providing further indirect evidence for a contribution of autoantibodies to disease pathology (432). The strongest evidence for a critical role of the humoral immune system in RA stems from preclinical model systems, which have permitted studying the complex disease mechanisms involved in the molecular and cellular processes ultimately resulting in joint inflammation and bone destruction (433). In these studies, the passive transfer of serum from mice with inflammatory arthritis into healthy animals was sufficient for induction of joint inflammation and bone destruction.

**Figure 12:**
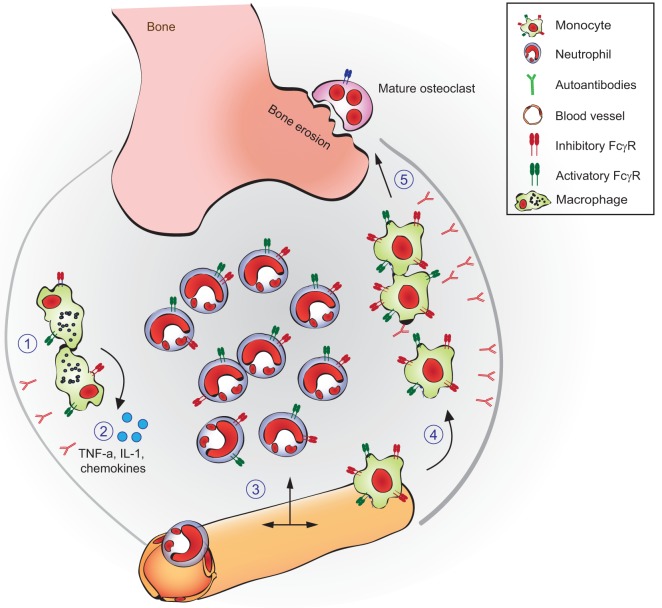
**Autoantibody-dependent pathology in inflammatory arthritis**. Shown are the autoantibody-driven processes that contribute to inflammation and bone destruction in inflammatory arthritis. Upon autoantibody deposition in the joints (1) tissue resident macrophages or mast cells become activated *via* Fcγ-receptors and release pro-inflammatory cytokines (including activated complement components) and chemokines (2). This leads to the recruitment of neutrophils and classical monocytes from the blood, leading to full-blown joint inflammation (3). The cytokine milieu favors the fusion of classical monocytes and their differentiation into immature osteoclasts (4). Immune complexes present in the joint bind to immature osteoclasts *via* FcgRs, which enhances their maturation into mature bone resorbing osteoclasts (5). Refer to the text, for a more detailed description.

Using a range of knockout mouse strains deficient in Fcγ-receptors or components of the complement pathway has helped to elucidate critical mechanisms responsible for triggering inflammation. Of note, both the complement pathway and cellular Fc-receptors were shown to participate in autoantibody-dependent inflammation and bone destruction (434). With respect to the involvement of complement, not the classical antibody-dependent pathway but rather non-classical pathways were shown to be essential for tissue inflammation, quite similar to what was observed for autoantibody-mediated skin blistering diseases (434). With respect to Fc-receptors, a hierarchy of the involvement of different receptors and cell types has emerged (Figure 12). Immediately after autoantibody injection, a mast cell-dependent opening of the vasculature was observed, which was abrogated in FcγR but not in complement-deficient animals (435, 436). Similar to the human disease, IL-1β, IL-6, and TNFα were suggested to be crucial for initiation and maintenance of inflammation (437). Interestingly, IL-1 secreted by mast cells was suggested as a key for initiation of inflammation (438). Apart from mast cells, tissue-resident macrophages were also suggested to be a key component during the very early phase of inflammation (439). Establishment and maintenance of inflammation is driven by neutrophils *via* activating Fc-receptors through activated complement components C3a and C5a (Figure 9). More recently, several studies have documented a critical role for Fc-receptors in the process of bone destruction (440–443). Consistent with the essential role of neutrophils and activating FcγRs in disease pathology, Syk or PLCg-deficient mice or animals with a neutrophil-specific deletion of Syk were protected from arthritis development (444, 445).

During chronic joint inflammation, osteoclasts differentiation from monocyte precursor cells recruited to the joint is favored by the pro-inflammatory milieu, which is the key event ultimately resulting in excessive bone resorption. It was demonstrated that especially inflammatory monocytes, which are recruited *via* the chemokine CCR2, differentiate into osteoclasts and are responsible for bone destruction (441). Moreover, autoantibodies modulated this process by binding to activating FcγRs expressed on immature and mature osteoclasts. Demonstrating the therapeutic potential of this approach, depletion of inflammatory monocytes or blocking autoantibody binding to osteoclast FcγRs diminished bone destruction (441). Moreover, characteristic changes in (auto)antibody glycosylation have been noted in mice and humans with arthritis (446, 447). Especially pro-inflammatory IgG glycoforms with low levels of terminal sialic acid and galactose residues, which interact efficiently with activating FcγRs were shown to dominate during active disease. More recent studies suggest that not only the enhanced triggering of pro-inflammatory effector functions but also the lack of anti-inflammatory sialic acid rich IgG glycovariants may contribute to the induction of excessive inflammation (448). Thus, infusion of IVIGs pooled from thousands of donors (IVIG therapy) was shown to induce resolution of inflammatory arthritis and of other autoantibody-dependent autoimmune diseases by restoring highly sialylated IgG glycovariants (400). As at least one part of the mechanism, an upregulation of the inhibitory FcγRIIb on joint infiltrating innate immune effector cells was shown which would increase the threshold for cell activation *via* autoantibody immune complexes (449). Ultimately this would reduce the downstream recruitment of neutrophils and osteoclast precursor cells from the blood and result in resolution of inflammation (448).

Taken together, activating FcγRs and activated complement components have been demonstrated to be of critical importance during all phases of autoantibody-dependent joint inflammation and may therefore represent promising therapeutic targets. Potential strategies may include blocking autoantibody interaction with activating FcγRs or inhibiting their downstream activating signals *via* small molecules. Alternatively, lowering autoantibody half-life by blocking antibody access to the neonatal Fc-receptor (FcRn) may also help to reduce inflammation and tissue destruction (450). In addition, a more in depth understanding of pathways essential for initiating resolution of inflammation (as induced by IVIG for example) may identify further treatment options to stop autoantibody pathology (448). As a most straightforward approach, IVIG variants with enhanced levels of anti-inflammatory IgG glycovariants may help to obtain enhanced therapeutic activity (451).

### Autoantibody-Induced Inflammation of the Optic Nerve and Spinal Cord: Neuromyelitis Optica Spectrum Disorder (NMOSD)

#### NMOSD: Epidemiology, Clinical Presentation, and Treatment Challenges

There is a rare neurological disorder caused by autoantibodies directed against a membrane protein expressed on astrocytes. The disease, NMO, recently renamed NMOSD, is an inflammatory demyelinating disease of the central nervous system that primarily affects optic nerves and spinal cord, and to a lesser extent brain (452, 453). The major clinical manifestations of NMOSD include recurrent bouts of eye and back pain with visual and motor impairment that can lead to blindness, paralysis, and death. Some patients with brain involvement manifest intractable vomiting, hiccups, and other symptoms. The prevalence of NMOSD is ~1–8 per 100,000 individuals, with a female:male ratio ~8:1 and a median age of presentation of 30–40 years, though the disease can present in the children and the elderly (454, 455). NMOSD was originally thought to be a subtype of multiple sclerosis, another inflammatory demyelinating disease of the central nervous system, but it is now clear that the diseases have distinct clinical signs and symptoms, pathogenesis, and responses to therapeutics. The mainstay of NMOSD therapy includes immunosuppression, plasma exchange, and B-cell depletion therapy, though new therapeutics are in the pipeline as discussed below. The current therapies are reasonably good, particularly B-cell depletion with rituximab, though many patients continue to have exacerbations and neurological deficit even with multiple drugs, and immunosuppressants can have significant side effects with long-term use.

#### NMOSD Pathogenesis

The major breakthrough in NMOSD was the discovery that the majority of patients have a circulating IgG1 autoantibody directed against AQP4, a water channel expressed at the plasma membrane of astrocytes throughout the central nervous system, including spinal cord, optic nerve, and brain (456). AQP4 is also expressed in various peripheral tissues including stomach, kidney, airways, and skeletal muscle. AQP4 functions a bidirectional water channel that facilitates water movement across cell plasma membranes in response to osmotic gradients produced by solute transport. Phenotype studies in AQP4 knockout mice have demonstrated its involvement in brain water movement, neuroexcitatory phenomena, and astrocyte migration (457). For example, mice lacking AQP4 are partially protected from cytotoxic (cell swelling) brain edema in stroke and other brain injuries (458). While most NMOSD patients are seropositive for anti-AQP4 autoantibodies (called AQP4-IgG), a small subset of patients are seropositive for anti-MOG (myelin oligodendrocyte glycoprotein) antibodies, and other patients show no detectable antibodies against AQP4 or MOG, though it is recognized that assay sensitivity remains imperfect and antibody titers vary over time.

**Figure 13:**
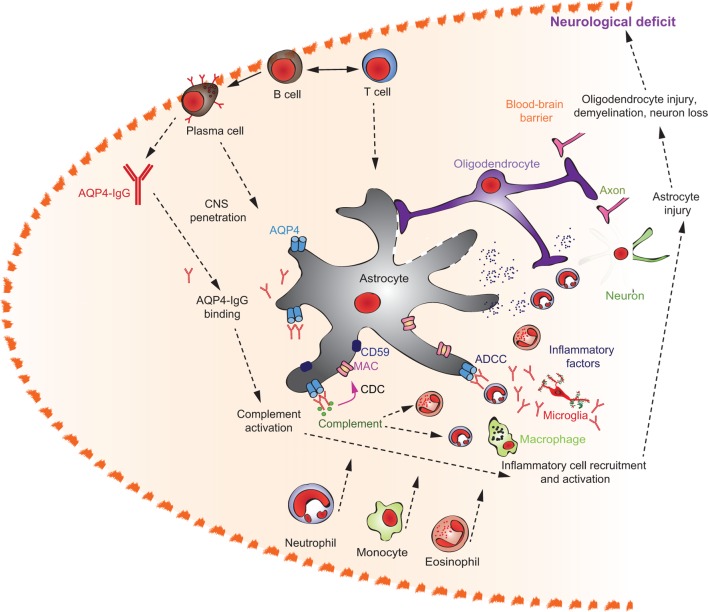
**Neuromyelitis optica spectrum disorder pathogenesis mechanisms**. Scheme shows astrocyte aquaporin-4 (AQP4) as the target of AQP4-IgG autoantibodies. Antibody binding initiates complement and cell-mediated cytotoxicity and an inflammatory response, resulting in oligodendrocyte injury and demyelination. Abbreviations: CDC, complement-dependent cytotoxicity; ADCC, antibody-dependent cellular cytotoxicity.

The major pathological features of NMOSD lesions include astrocyte damage with loss of AQP4 and glial fibrillary acidic protein, inflammation with prominent granulocyte and macrophage infiltration, vasculocentric deposition of activated complement, and demyelination; in later stages, there is neuronal loss and scarring. The NMOSD pathogenesis mechanism in AQP4-IgG seropositive patients does not involve antibody effects on AQP4 function, but rather primary damage to astrocytes by complement- and cell-mediated mechanisms, as shown in Figure 13. Evidence from human pathological specimens and rodent models of passive AQP4-IgG transfer suggests that binding of AQP4-IgG to AQP4 on astrocytes initiates complement-dependent cytotoxicity (CDC) and antibody-dependent cellular cytotoxicity (ADCC), astrocyte damage, inflammation, and BBB disruption, which leads to oligodendrocyte injury and demyelination (452, 453). Complement activation plays a central role in NMOSD pathology, both by formation of a terminal attack complex on astrocytes, as well as by elaboration of anaphylatoxins that attract and activate inflammatory leukocytes. There may also be involvement of AQP4-sensitized T-cells (459), though it is not clear whether T-cells are involved in permeabilization of the BBB and/or in astrocyte cytotoxicity (460). Major, largely unanswered, questions in NMOSD pathogenesis include why disease is localized mainly to spinal cord and optic nerve without involvement of peripheral, tissues expressing AQP4, how early oligodendrocyte damage is caused by AQP4-IgG binding on astrocytes, and how peripherally generated AQP4-IgG enters the central nervous system to initiate disease.

#### Emerging Treatments and Novel Therapeutic Targets

Several NMOSD therapeutics in the development pipeline, many of which emerged from the improved understanding of pathogenesis (461, 462). Given the central role of complement, the C5 inhibitor Eculizumab is in clinical trials following encouraging data in a small open-label study in AQP4-IgG seropositive patients having frequent disease exacerbations (113). Anti-IL6 receptor antagonists that target antibody-producing plasma cells are in clinical studies. The repurposing of other approved drugs for NMOSD is under evaluation, including antibodies targeting VEGF and CD-19/20, small molecule drugs targeting neutrophils and eosinophils, and IVIG. Therapeutics in preclinical development include antibodies (aquaporumab) targeting AQP4-IgG binding to AQP4 (463), and AQP4-IgG inactivation by enzymatic deglycosylation (464). Other potential targets for NMO therapeutics include AQP4 cell surface expression and its supramolecular aggregation, complement inhibitor protein CD59, and various components of the CDC, ADCC, and inflammation pathways. Recently, experimental animal data support the possibility of a remyelination approach in NMOSD to limit neuron loss (465). Lastly, the possibility of antigen-specific tolerization against AQP4 is a theoretical possibility, as is autologous hematopoietic stem cell transplantation.

## Diagnostic Tools for Detection of Autoantibodies

### Indirect Immunofluorescence

**Figure 14:**
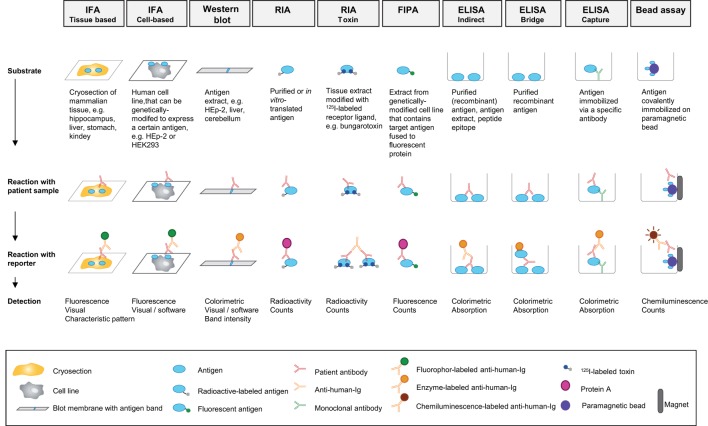
**Detection of autoantibodies**. Details are explained in the text. Abbreviations: ELISA, enzyme-linked immunosorbent assay; FIPA, fluorescence immunoprecipitation assay; IFA, indirect immunfluorescence assay; RIA, radioimmunoassay.

One of the “gold standards” for autoantibody determination maintains to be the indirect immunofluorescence assay (IFA) using cryosections of mammalian tissue or cultured cell lines, e.g., HEp-2, which is the standard substrate for the screening of antinuclear antibodies (Figure 14). The immunoassay principle essentially consists of two steps: in the initial incubation a human sample like blood or CSF is brought into contact with the substrate. Subsequently, unbound sample constituents are washed off and the bound antibodies are visualized with fluorochrome-labeled secondary antibodies. The evaluation is done by fluorescence microscopy leading to the interpretation of the staining patterns. The approach has three main advantages: (i) all autoantigens in a given substrate are present and displayed in their native environment, (ii) it is possible to screen for antibodies against unidentified autoantigens based on characteristic staining patterns such that even *de novo* screenings of patient cohorts are possible, and (iii) a negative outcome rules out the presence of multiple autoantibody specificities with a single analysis. The concentration of the respective autoantibody can be estimated by titering-out the sample. However, in some cases of autoantibodies against soluble antigens or against those that are prone to denaturation by necessary fixation steps, the use of IFA is limited. Lately, the introduction of automated digitalization together with the computer-assisted evaluation of the results (466–468) and the development of genetically modified cells expressing the necessary autoantigens—often designated as CBA—have rejuvenated the use of IFA (456, 469–475). Another support for the use of IFA as a routine diagnostic platform is the availability of incubation automates that decrease hands-on time and lower the total costs per analysis.

### Immunoprecipitation

Due to the highly sensitive detection of radioactive substances, radioimmunoassays (RIAs) based on specifically labeled antigens were developed decades ago and are still a good option for the detection of autoantibodies against membrane proteins. Commonly, a radiolabel like 125-iodine is chemically or enzymatically attached to a purified ligand, e.g., 125-I-α-bungarotoxin for the labeling of AChR (476). The ligand is then incubated with tissue or cell extracts containing the desired membrane protein in a conformation that is able to specifically bind the ligand. Alternatively, *in vitro*-translated 35-S-methionine labeled antigens can be used with the general limitation to simple (intracellular) antigens (477). The radiolabeled antigen is then brought into contact with a human sample and the immune complexes formed in this step as well as free Igs are precipitated by a secondary antibody or an affinity matrix like agarose-bound staphylococcal protein G. The amount of precipitated radioactivity is a quantitative measure of the antibody against the labeled target antigen in the sample. Classically, RIAs have been used for the sensitive detection of autoantibodies against both intracellular and surface-exposed target antigens, e.g., against double-stranded DNA in SLE or the nicotinic AChR in MG. As an alternative to the radiolabel, fluorophores can be introduced to the target antigen, either by chemical labeling of the isolated target antigen or by its recombinant expression in frame with a fluorescent fusion protein, e.g., a variant of the green fluorescent protein (478).

### ELISA, Fluorescence Immunoassay (FIA), Chemilumenescence Immunoassay (CLIA), and Bead-Based Assays

Another classical method for the semiquantitative determination of autoantibodies is the ELISA. Basically, a target antigen isolated from a native biosource or a recombinant host by a combination of biochemical procedures is initially immobilized on a solid phase, e.g., in cavities of plastic microplates or on the surface of membranes. The immobilization allows for sophisticated blocking, washing, and stabilization steps. The sample to be investigated is eventually brought into contact with the target antigen, unbound sample constituents are removed by washing and bound antibodies are detected with the help of a secondary antibody coupled to a reporter. Microplate ELISA typically makes use of reporting enzymes that convert a colorless reagent to a chromophore that is subsequently quantified with a spectrophotometer. Membrane-based ELISA (commonly designated “lineblot” or “dot blot”) instead commonly employ a precipitating chromophore that can be quantified by densitometric analysis of electronic images produced with the help of a flatbed scanner or a camera system (479). Alternative protocols are based on reagents that produce a chemilumenscent reaction upon conversion by the reporter enzyme in a CLIA or make use of a fluorophore-reporter in FIA, in some cases combined with fluorescent beads in the form of addressable laser-bead immunoassays (ALBIA) (480, 481). Alternatively, paramagnetic microparticles were developed as an additional solid phase that allows for magnetic separation of the target antigen from other reagents in subsequent steps. This bead-based assay platform is in most cases combined with chemilumenescence detection (482). All of the different methods and platforms can be partnered with sophisticated incubation, handling and detection devices or are integrated into commercially available test systems that allow for semi- or fully automated incubation and evaluation of the samples resulting in comprehensive output.

### Western Blot

A further classical method for qualitative autoantibody determination is the Western blot (482). For this method, tissue or cell extracts containing one or several target antigens are separated by gel electrophoresis and subsequently transferred to membranes. The sample that is to be investigated for antibodies is then incubated and bound antibodies are visualized with the help of a secondary antibody coupled to a reporter, e.g., an enzyme converting a colorless reagent to a precipitating chromophore. Generally, the denaturing effect of the electrophoresis limits the determination of antibodies to those binding denaturation-insensitive structures (“linear epitopes”).

### Multiplexing

In most cases, screening and confirmation assays are combined for autoantibody determination to achieve a meaningful result with regard to the clinical problem. Modern test systems combine both approaches in one. Slides that carry more than one substrate per incubation field enable multiplexed testing in the IFA format. For example, the combination of different tissues like rat kidney, liver, and stomach—as commonly employed for the determination of antibodies associated with autoimmune liver diseases—allows for a more differentiated, highly specific evaluation with just one analysis. The biochip format made of mosaics (471, 483–485) of millimeter-sized glass chips coated with the diagnostically relevant antigenic substrates even allows the combined use of tissue cryosections, cultured (genetically modified and unmodified control) cells, and directly immobilized purified antigens for a comprehensive analysis covering the whole spectrum of autoantibodies relevant for differential diagnostics of autoimmune syndromes. Moreover, microplate and membrane-based ELISA as well as ALBIA can be used to form antigen profiles for the detection of multiple antibodies in parallel. Multiparametric lineblots have been developed for this purpose carrying more than 20 individually optimized membrane chips on a plastic layer and harboring antigen mixtures, individual antigens and even lipids (486–488).

It should be emphasized that none of the above approaches were used to discover TSHR autoantibodies nor can they be used clinically to measure TSHR antibodies in Graves’ patients. In 1956, using guinea pigs as a read-out, Graves sera were found to contain a thyroid-stimulating factor with a duration of action more prolonged than TSH [“long-acting thyroid stimulator” (LATS)] (489). LATS was later found to be an IgG molecule. After the discovery of the TSHR, it was shown that LATS (like TSH) activated thyrocyte adenylyl cyclase and competed for TSH binding to the TSHR. This background to TSHR antibodies suggests that, like Graves’ patients, some patients described as seronegative for a particular autoantibody may have functional antibodies not detectable by the conventional approaches listed above.

## Conclusion

As highlighted herein, autoantibodies may cause pathology through a wide range of mechanisms. Insights into these pathways identified several novel therapeutic targets, such as FcγR-induced neutrophil activation, which go beyond unspecific immunosuppression and are already in clinical trials (340). Preclinical models even point toward the possibility to specifically target autoantibody-specific B cells and/or plasma cells, which could be considered as curative treatment (117). Based on the vast amount of insights into pathomechanism of autoantibody-induced pathology, some of which have been reviewed herein, we expect that these concepts will be evaluated in clinical trials, which will significantly improve the still not satisfying therapeutic options currently used to treat autoimmunity.

## Author Contributions

All authors listed, have made substantial, direct, and intellectual contribution to the work, and approved it for publication.

## Conflict of Interest Statement

RL received speakers honoraria from and/or has consultancy agreements with AbbVie, Allmiral, ArGEN-X, Biogen Idec, Biotest, Novartis, UCB Phrama, Topadur, and TxCell and holds the patent “Soluble Fc gamma receptor for treatment of autoimmune bullous diseases” (US 15/029,994). FL has received speakers’ honoraria from Grifols, Biogen, Teva, and Roche. His institution offers commercial antibody testing, and he does not receive personal benefits from this. All other authors declare no conflicts of interest. CP and LK are employees of the Euroimmun AG, a company that develops, manufactures, and markets human diagnostics. JL and JML have a patent application for an antigen-specific immunosuppressive therapy for MG.
